# Role of obesity in smoking behaviour: Mendelian randomisation study in UK Biobank

**DOI:** 10.1136/bmj.k1767

**Published:** 2018-05-16

**Authors:** Robert Carreras-Torres, Mattias Johansson, Philip C Haycock, Caroline L Relton, George Davey Smith, Paul Brennan, Richard M Martin

**Affiliations:** 1 Section of Genetics, International Agency for Research on Cancer, Lyon, France; 2 Medical Research Council Integrative Epidemiology Unit, School of Social and Community Medicine, University of Bristol, Bristol, UK; 3 Department of Population Health Sciences, Bristol Medical School, University of Bristol, Bristol, UK; 4 National Institute for Health Research Biomedical Research Centre at University Hospitals Bristol NHS Foundation Trust and the University of Bristol, Bristol, UK

## Abstract

**Objective:**

To determine whether body mass index, body fat percentage, and waist circumference influence smoking status and intensity.

**Design:**

Mendelian randomisation study.

**Setting:**

UK Biobank, with replication of results from the Tobacco and Genetics (TAG) consortium.

**Participants:**

European descent participants from the UK Biobank cohort (n=372 791) and the TAG consortium (n=74 035).

**Main outcome measures:**

Risk of current and past smoking, number of cigarettes smoked per day, age of smoking initiation.

**Results:**

The Mendelian randomisation analysis indicated that each standard deviation increment in body mass index (4.6) increased the risk of being a smoker (odds ratio 1.18 (95% confidence interval 1.13 to 1.23), P<0.001). This association was replicated in the TAG consortium data (1.19 (1.06 to 1.33), P=0.003). Furthermore, each standard deviation increment in body mass index was estimated to increase smoking intensity by 0.88 cigarettes per day (95% confidence interval 0.50 to 1.26, P<0.001) in UK Biobank and 1.27 cigarettes per day in the TAG consortium (0.46 to 2.07, P=0.002). Similar results were also seen for body fat percentage and waist circumference in both UK Biobank and the TAG consortium data.

**Conclusions:**

These results strongly suggest that higher adiposity influences smoking behaviour and could have implications for the implementation of public health interventions aiming to reduce the prevalence of these important risk factors.

## Introduction

Obesity and tobacco smoking are important risk factors for a wide variety of non-communicable diseases,^1^ but their inter-relationship is complex and not well understood. Observational studies consistently show an inverse association between current cigarette smoking and body weight, followed by weight gain after smoking cessation.^2^ ^3^ ^4^ ^5^ ^6^ The first association is thought to be an effect of smoking on reducing appetite, and the second could be a consequence of higher caloric intake due to replacement of the smoking habit with food intake.^7^ ^8^ Paradoxically, smokers have been reported to present with higher waist circumference than never smokers.^9^ A positive correlation between body mass index and smoking intensity in both current and former smokers has also been reported.^2^ ^6^ These observations might be due to other lifestyle factors, because physical inactivity, unhealthy diet, and alcohol consumption are positively correlated with both adiposity and smoking parameters.^2^ ^4^ However, general and abdominal obesity could also increase the propensity to take up smoking, as well as increase smoking intensity. People who are obese might start smoking as a weight reduction strategy, or alternatively, obesity could enhance nicotine dependence and affect smoking intensity.^10^ Further understanding of the causal relation between obesity and smoking behaviour is important for clinical and public health initiatives that aim to reduce both risk factors, and could also inform aetiological research for diseases where obesity and smoking have been implicated.

Mendelian randomisation is an analytical approach that uses genetic markers of an exposure rather than the exposure itself, the major advantage being that germline genetic associations cannot be explained by reverse causation and are less susceptible to confounding.^11^ An association that is observed by Mendelian randomisation is therefore likely to reflect a causal relation.^12^ ^13^ ^14^ Genetic variants that are associated with body mass index, body fat percentage, and waist circumference can therefore be used as proxies to attain unconfounded estimates of the influence of obesity on smoking behaviour. Recent genome wide association studies revealed 77 genetic regions associated with body mass index, 12 with body fat percentage, and 45 with waist circumference, in large European based samples of more than 100 000 participants,^15^ ^16^ ^17^ providing robust instruments for use in our Mendelian randomisation analyses.

Initial genetic evidence suggests a possible common biological basis for nicotine addiction and obesity.^18^ In this study, we tested the hypothesis that obesity causally influences the risk of being a smoker and also of smoking intensity using a Mendelian randomisation framework based on 372 791 individuals from the UK Biobank cohort, and independent data from 74 035 individuals from the Tobacco and Genetics (TAG) consortium.

## Methods

Genetic variants that are strongly associated with adiposity parameters were identified on the basis of results from the largest genome wide association study published so far.^15^ ^16^ ^17^ These genetic variants were subsequently used as proxies for adiposity parameters and evaluated in relation to smoking parameters in the UK Biobank sample. Details of the methods and relevant study samples are provided below.

### UK Biobank sample

UK Biobank is a prospective cohort that recruited more than 500 000 men and women aged 40-96 years between 2006 and 2010, and collected anthropometric, health, and lifestyle data, as well as biological samples.^19^ Of 487 409 individuals who were genotyped in UK Biobank, we used data for 372 791 European descent participants with valid adiposity and smoking behaviour measures at recruitment. European background was genetically assessed through principal component analyses of data from genome wide association studies. Sample quality control steps are given in the supplementary methods.

### Obesity and smoking behaviour measures

#### Body mass index, body fat percentage, and waist circumference

Body mass index was calculated as weight divided by height squared (kg/m^2^). Standing height (cm) was measured by a Seca 202 device (SECA). Body fat percentage was estimated by bioelectrical impedance, with measures ranging from 1% to 75% in 0.1% increments. Weight and bioimpedance were measured by the Tanita BC-418MA body composition analyser (Tanita Corporation of America). Finally, waist circumference was manually measured in centimetres.

#### Smoking behaviour

Three smoking behaviour parameters were collected by questionnaire or interview at recruitment. We categorised smoking status as never, former, or current smokers. In former and current smokers (n=169 056), we also analysed the number of cigarettes smoked per day and age of smoking initiation. Cigarettes per day was included in the analysis if the answer was between one and 150, and we received a total of 113 295 valid answers. Age of smoking initiation (years) was log-transformed to reduce positive skew in the distribution, and null responses were rejected (that is, not answered or age was lower than 5 years or higher than current age), giving 119 939 valid answers.

### Statistical methods

#### Relation between direct adiposity measures and tobacco smoking in UK Biobank

To provide a baseline assessment of the relation between direct adiposity measures and smoking in UK Biobank, directly measured body mass index, body fat percentage, and waist circumference were evaluated in relation to risk of being a smoker by use of logistic regression (ever *v* never smokers, and former *v* current smokers), and in relation to smoking intensity (cigarettes smoked per day) and age of smoking initiation by use of linear regression. Models were adjusted for age, sex, and population stratification.

#### Genetic instruments for the adiposity exposures

We built genetic instruments for our exposure measures of interest (body mass index, body fat percentage, and waist circumference) using single nucleotide polymorphisms (SNPs). These SNPs were independently (linkage disequilibrium R^2^ measure <0.01) associated with body mass index, body fat percentage, or waist circumference measures (at P<5×10^−8^) in the largest European descendent genome wide association studies so far.^15^ ^16^ ^17^ After considering linkage disequilibrium, 73 independent SNPs were maintained as instruments for body mass index,^15^ 12 SNPs for body fat percentage,^16^ and 44 for waist circumference.^17^

We extracted these genetic variants from the UK Biobank imputed dataset to calculate genetic scores for body mass index, body fat percentage, and waist circumference by summing up the increasing-trait allele dosages weighted by their relative effect size on adiposity parameters (β_GP_), as reported in the original genome wide association studies^15^ ^16^ ^17^ (Σ_i_ dosage_GPi_×β_GPi_). SNPs with ambiguous strand codification (adenine/thymine or cytosine/guanine) were replaced by SNPs in tight genetic linkage (R^2^ >0.8) using the proxysnps R package (European populations; Bioconductor) or removed from the analyses. All SNPs had an imputation quality score (R^2^) higher than 0.95. The SNP effects (β_GP_) used to build the genetic scores were originally scaled according to a standard deviation increment (SD_X_) of the adiposity trait in the discovery study (SD_body mass index_=4.6; SD_body fat percentage_=6.6%; SD_waist circumference_=12.2 cm); hence, a one unit increment in the genetic score would indicate one standard deviation increase in the adiposity phenotypes according to the discovery sample. 

The fraction of variance in the phenotype explained by the SNPs in the discovery study was 2.4% for body mass index,^15^ 0.6% for body fat percentage,^16^ and 1.6% for waist circumference.^17^ To evaluate potential pleiotropic effects by socioeconomic factors, we also re-evaluated associations by excluding SNPs nominally associated (P<0.05) with the Townsend deprivation index in UK Biobank. These sensitivity criteria resulted in the exclusion of 14 SNPs from the body mass index instrument, two from the body fat percentage instrument, and nine from the waist circumference instrument. Supplementary table A shows SNP summary statistics describing their association with adiposity parameters (β_GP_ and standard errors (SE_GP_)) from the original genome wide association studies, with Townsend deprivation index from UK Biobank samples, and SNP imputation quality parameters in UK Biobank. For body mass index, we also evaluated a subset of 40 SNPs that were reported to cluster in at least one neuronal related biological pathway (neuronal developmental processes, neuronal expression, neurotransmission or hypothalamic expression, and regulatory function).^15^

#### Power assessment

Power estimation for the Mendelian randomisation analyses were performed on the basis of the phenotype variance explained by the genetic instruments and the analysed sample size.^20^ We observed sufficient power (>80%) to detect a risk increase for being a smoker of 1.13 for the body mass index instrument, 1.18 for the waist circumference instrument, and 1.31 for the body fat percentage instruments in both the UK Biobank and TAG study samples separately (supplementary fig A-I), with similar results available for former smokers (supplementary fig A-II). The minimum number of extra cigarettes smoked per day that our analyses were able to detect using the genetic instruments for body mass index, waist circumference, and body fat percentage were 0.09, 0.11 and 0.19, respectively (supplementary fig A-III). And finally, we had enough power to detect differences in log-transformed years of age of smoking initiation of 0.11, 0.14, and 0.24 using the body mass index, waist circumference, and body fat percentage genetic instruments, respectively (supplementary fig A-IV).

#### Estimation of the effect of obesity on smoking behaviour by use of a genetic score

We estimated the influence of obesity on smoking behaviour by including the genetic scores for body mass index, body fat percentage, or waist circumference as explanatory variables in logistic regression models (ever *v* never smokers, and former *v* current smokers) or linear regression models (cigarettes smoked per day, and age of smoking initiation), with smoking parameters as response variables. These regression models were adjusted for age, sex, genotyping array, and population stratification.

#### Sensitivity analyses

Sensitivity analyses were based on summary statistics for genetic associations included in supplementary table A. We used Mendelian randomisation-Egger weighted linear regression of the SNP-to-smoking effect estimates (β_GD_) on the SNP-to-obesity effect estimates (β_GP_)^21^ to detect overall directional pleiotropy biasing our initial risk estimates, and SNPs behaving as genetic outliers (supplementary methods provides more details). The corresponding funnel plots, showing the contribution of the SNP ratio estimates (β_GDi_/β_GPi_) to the Mendelian randomisation estimates, were generated by use of the ggplot2 R package (R Project; supplementary methods). Additional sensitivity analyses based on the distribution of SNP ratio estimates (β_GDi_/β_GPi_) were performed, namely the weighted median approach^22^ and the modal based estimate approach.^23^ These methods are less sensitive to the effect of pleiotropic variants behaving as outliers, and to the presence of invalid instruments (supplementary methods). Finally, to identify individual SNPs strongly influencing the association estimates, we obtained Mendelian randomisation estimates leaving out one SNP at a time from the instrumental variable and plotting the resulting effect estimates in a histogram. Individual SNPs that strongly influence the overall effect estimate would be reflected with a deviating effect estimate. We also repeated the analyses on the UK Biobank sample separately by sex, and tested the between-sex heterogeneity (supplementary methods).

### Replication of the results

To validate the estimated causal effects of obesity on smoking behaviour obtained from the UK Biobank sample, independent summary statistics of the obesity SNPs for smoking behaviour from genome wide association studies were obtained from the TAG consortium^24^ through MR-Base (www.mrbase.org/),^25^ an online platform for Mendelian randomisation analyses. The TAG data included the same smoking behaviour parameters as the UK Biobank sample, including smoking status (ever *v* never (n=74 035) and former *v* current (n=41 278)), cigarettes per day (n=38 181), and log-transformed age of smoking initiation (n=24 114; supplementary table A). The SNP summary data for adiposity parameters were from the original genome wide association study where these SNPs were identified, similar to the UK Biobank data analyses. We used a likelihood based approach^26^ to perform Mendelian randomisation analyses based on summary data for the smoking behaviour parameters for obesity SNPs (supplementary methods). Sensitivity analyses were performed as described for UK Biobank data.

### Patient involvement

Patients and service users were not involved in setting the aims and design of the study, nor the analyses or the interpretation of the results.

## Results

### Baseline characteristics of UK Biobank sample and the relation between direct adiposity measures and smoking behaviour in UK Biobank

The UK Biobank sample comprised 53.7% women (table 1), and the median age at recruitment was 58.0 years (interquartile range 51.0-63.0). The distribution of adiposity and smoking behaviour variables in the UK Biobank sample are described in table 1 and table 2. As observed in previous studies, current smokers had a lower body mass index than never smokers (−0.22 (95% confidence interval −0.27 to −0.16)). Conversely, former smokers had a higher body mass index than current smokers (1.04 (0.98 to 1.09)).

**Table 1:** Sample characteristics of body size parameters by smoking and sex categories in UK Biobank. Data are mean (standard deviation)

Body size parameters	Total (n=372 791)	Smoking category				Sex	
		Never (n=203 735)	Former (n=131 537)	Current (n=37 519)		Female (n=200 247)	Male (n=172 544)
Body mass index	27.4 (4.8)	27.1 (4.7)	28.0 (4.7)	27.0 (4.8)		27.0 (5.1)	27.9 (4.2)
Weight (kg)	78.3 (15.9)	77.0 (15.6)	80.5 (16.0)	78.0 (16.3)		71.5 (13.9)	86.2 (14.3)
Height (cm)	168.8 (9.2)	168.3 (9.3)	169.4 (9.1)	169.5 (9.2)		162.7 (6.2)	175.9 (6.7)
Waist circumference (cm)	90.4 (13.5)	88.8 (13.2)	92.6 (13.6)	91.2 (13.5)		84.6 (12.5)	97.1 (11.3)
Body fat percentage (%)	31.4 (8.5)	31.5 (8.6)	31.7 (8.2)	29.9 (8.6)		36.6 (6.9)	25.3 (5.8)

**Table 2:** Sample characteristics of smoking parameters by body mass index and sex categories in UK Biobank ever smokers (current plus former smokers). Data are mean (standard deviation)

Smoking parameters	Total (n=169 056)	Body mass index category						Sex	
		Underweight (<18.5; n=816)		Normal (18.5-25.0; n=49 017)	Overweight (25.0-30.0; n=74 439)	Obese (>30.0; n=44 784)		Female (n=81 091)	Male (n=87 965)
Age started smoking (years)	17.3 (4.2)	17.5 (4.8)		17.6 (4.2)	17.3 (4.2)	17.1 (4.3)		17.8 (4.4)	16.9 (4.0)
No of cigarettes smoked per day									
Ever smokers	18.4 (10.1)	16.6 (10.5)	15.9 (8.6)		18.2 (9.6)	21.1 (11.5)		16.1 (8.2)	20.5 (11.2)
Current smokers*	15.8 (8.4)	16.8 (11.1)	15.0 (8.2)		15.6 (8.1)	17.3 (9.0)		14.2 (7.3)	17.4 (9.2)

* N=37 519 current smokers.

**Fig 1:**
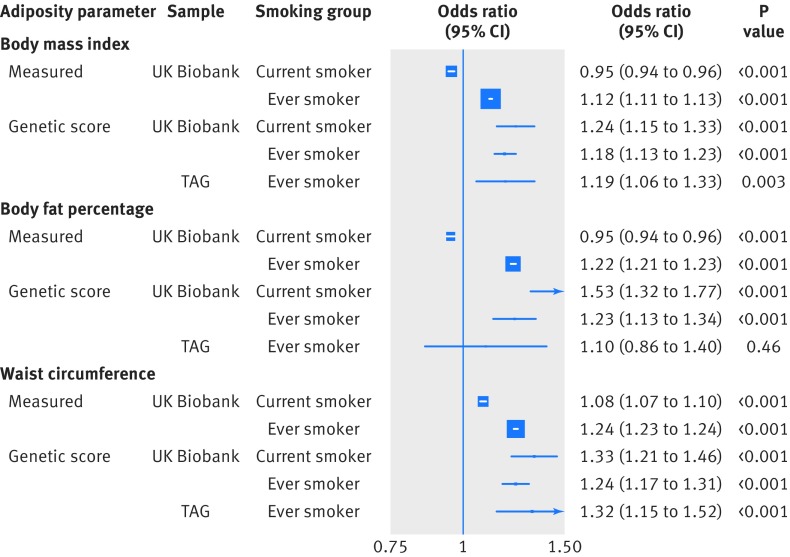
Association between measured and genetically determined increase in one standard deviation in adiposity parameters and smoking status (ever *v* never smokers). TAG=Tobacco and Genetics consortium

**Fig 2:**
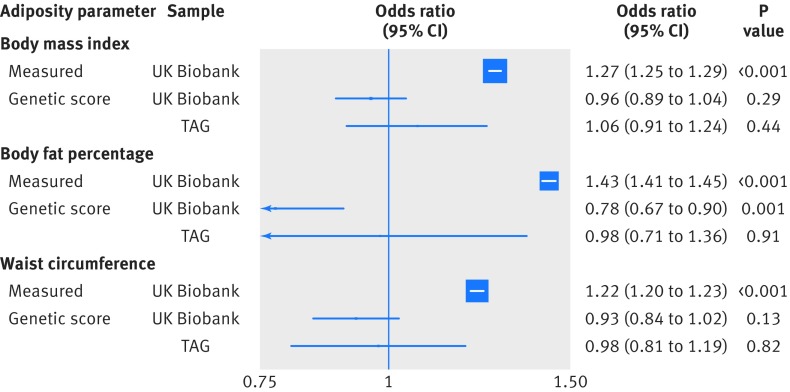
Association between measured and genetically determined increase in one standard deviation in adiposity parameters and smoking cessation (former *v* current smokers). TAG=Tobacco and Genetics consortium

**Fig 3:**
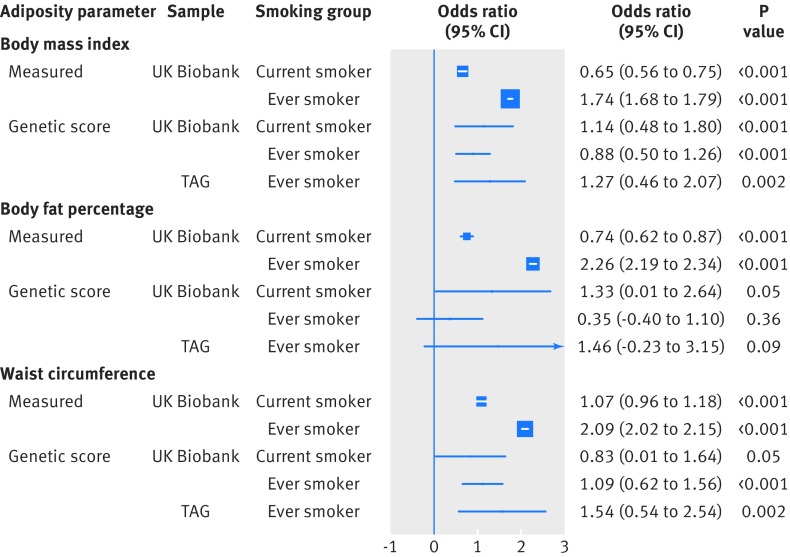
Association between measured and genetically determined increase in one standard deviation in adiposity parameters and number of cigarettes smoked per day. TAG=Tobacco and Genetics consortium

**Fig 4:**
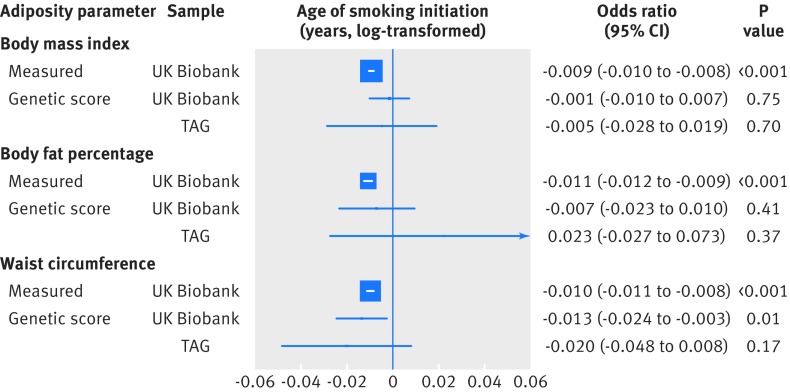
Association between measured and genetically determined increase in one standard deviation in adiposity parameters and age (years) of smoking initiation (log-transformed). TAG=Tobacco and Genetics consortium. One year equals to about 0.06 units of log-transformed years. Genetic score results reflected the estimated effects after single nucleotide polymorphisms nominally associated with the Townsend deprivation index were removed from the genetic instruments

When considering the risk of smoking in response to direct adiposity measures, each standard deviation increase in measured body mass index (4.6) was associated with a lower risk of being a current smoker (odds ratio 0.95 (95% confidence interval 0.94 to 0.96)), compared with being a never smoker (fig 1). However, each standard deviation increase was also associated with a higher risk of being an ever (current and former) smoker (1.12 (1.11 to 1.13)), compared with being a never smoker (fig 1). Furthermore, higher measured body mass index, body fat percentage, and waist circumference were associated with an increased risk of being a former smoker compared with being a current smoker (all P<0.001; fig 2). Additionally, each standard deviation increase in directly measured body mass index was associated with smoking intensity in current smokers (0.65 extra cigarettes smoked per day (95% confidence interval 0.56 to 0.75)) and in ever smokers (1.74 extra cigarettes (1.68 to 1.79); fig 3). Finally, higher body mass index was associated with smoking initiation at younger ages (by about two months earlier per standard deviation increase of body mass index; fig 4). Body fat percentage and waist circumference showed similar associations with smoking behaviour parameters as those for body mass index (fig 1, fig 2, fig 3, and fig 4).

### Validity of genetically determined adiposity measures in predicting measured adiposity in UK Biobank

Each unit increase in the genetic scores reflected one standard deviation of measured phenotypes. A one unit increase in the body mass index genetic score was associated with a 4.0 (95% confidence interval 3.9 to 4.1) increase in measured body mass index in the UK Biobank and explained 1.6% of measured body mass index variance. This relation was slightly stronger in women (4.2 (4.0 to 4.3)) than in men (3.9 (3.7 to 4.0); P_heterogeneity_=0.003). Similarly, the genetic score for body fat percentage was strongly associated with measured body fatness (5.1% per unit of increase in the genetic score of body fat percentage (95% confidence interval 4.8% to 5.3%; 0.2% of measured variance of body fat percentage). This relation was slightly stronger in women (5.5% (5.1% to 5.9%)) than in men (4.6% (4.2% to 5.0%); P_heterogeneity_=0.002). 

Finally, one unit of increase in the genetic score of waist circumference corresponded to 10.1 cm of waist circumference (95% confidence interval 9.7 to 10.4; 0.9% of measured variance of waist circumference), but no sex differences were observed (P_heterogeneity_=0.67). These estimates can be compared with the standard deviation observed for obesity parameters in the original discovery studies that defined the respective genetic instruments (4.6 for body mass index, 6.6% for body fat percentage, and 12.2 cm for waist circumference).

### Association between genetically determined adiposity and smoking behaviour

#### Smoking status

By contrast with the analysis using directly measured body mass index, each genetically predicted standard deviation increase in body mass index showed a similar risk increase of being a current smoker (odds ratio 1.24 (95% confidence interval 1.15 to 1.33) or ever smoker (1.18 (1.13 to 1.23); fig 1). Similarly, one standard deviation increase in predicted body fat percentage was positively associated with being a current smoker (1.53 (1.32 to 1.77)) or ever smoker (1.23 (1.13 to 1.34); fig 1). The genetic score for waist circumference was also positively associated with being a current smoker (1.33 (1.21 to 1.46)) or ever smoker (1.24 (1.17 to 1.31); fig 1). The associations of genetically predicted body mass index and waist circumference with risk of being a smoker were replicated in the TAG data (1.19 (1.06 to 1.33) and 1.32 (1.15 to 1.52), respectively; fig 1). Sensitivity analyses provided little evidence for genetic outliers, particularly influential SNPs, or directional pleiotropy. However, a funnel plot and density function indicated heterogeneity among SNP estimates, especially for body mass index and waist circumference (P_SNP-heterogeneity_<0.01; supplementary figs B-D).

#### Smoking cessation

There was no association between the genetically determined body mass index or waist circumference and the odds of quitting smoking in UK Biobank (odds ratio 0.96 (95% confidence interval 0.89 to 1.04) and 0.93 (0.84 to 1.02), respectively) or in the TAG study (1.06 (0.91 to 1.24) and 0.98 (0.81 to 1.19), respectively; fig 2). A standard deviation increment in body fat percentage was associated with a lower odds of quitting smoking (0.78 (0.67 to 0.90)), but this association was not replicated in the TAG study (0.98 (0.71 to 1.36)). Sensitivity analyses did not reflect any bias in the initial risk estimates (P*_Intercept_* >0.03; supplementary figs E-G).

#### Number of cigarettes smoked per day

Each genetically predicted standard deviation increase in body mass index was positively associated with smoking intensity in current smokers (1.14 extra cigarettes smoked per day (95% confidence interval 0.48 to 1.80)) and ever smokers (0.88 (0.50 to 1.26); fig 3). A similar association was also observed for waist circumference (0.83 (0.01 to 1.64) in current smokers and 1.09 (0.62 to 1.56) in ever smokers; fig 3). These associations were replicated in TAG ever smokers (1.27 extra cigarettes (0.46 to 2.07) per standard deviation increase in body mass index and 1.54 (0.54 to 2.54) per waist circumference increase; fig 3). The body fat percentage genetic score did not appear initially associated with smoking intensity in the UK Biobank ever smokers (0.35 (−0.40 to 1.10)), nor in the TAG sample (1.46 (−0.23 to 3.15); fig 3). 

Sensitivity analyses in ever smokers did not detect pleiotropic effects biasing these initial estimates. However, the SNP representing the *FTO* gene region in the three genetic instruments (rs1421085 for body mass index and body fat percentage, and rs62048402 for waist circumference) had an important influence on the overall association estimate, and exclusion of this SNP suggested stronger associations between the genetic instruments and smoking intensity. In UK Biobank, the associated extra cigarettes smoked per day were 1.23 (95% confidence interval 0.82 to 1.65) per standard deviation increase in body mass index, 0.98 (0.08 to 1.88) per body fat percentage increase, and 1.64 (1.11 to 2.17) per waist circumference increase. In the TAG consortium, the extra cigarettes smoked per day were 1.36 (0.50 to 2.22) per standard deviation increase in body mass index, 1.62 (−0.23 to 3.50) per body fat percentage increase, and 1.82 (0.75 to 2.90) per waist circumference increase (supplementary figs H-J).

#### Age of smoking initiation

Initial analyses provided some evidence for a causal role of adiposity in affecting age at smoking initiation, with each standard deviation of higher body mass index, body fat percentage, and waist circumference associated with a younger age of smoking initiation (−0.01 log-transformed years (about two months; 95% confidence interval −0.02 to −0.002); −0.01 (−0.03 to 0.005); and −0.02 (about four months; −0.03 to −0.01); respectively). However, these associations were not replicated in the TAG sample (−0.01 (−0.03 to 0.01); 0.01 (−0.04 to 0.05); and −0.02 (−0.04 to 0.01); respectively). Sensitivity analyses did not reflect any bias in the initial risk estimates (supplementary figs K-M).

### Pleiotropy by social deprivation

We evaluated whether pleiotropy by social deprivation could have affected our results by rerunning the analyses after removing SNPs nominally associated with the Townsend deprivation index. The association estimates between obesity measures and smoking were largely unaffected (supplementary figs N-Q), with the exception of the analyses on age of smoking initiation. Each standard deviation increase in body mass index was not associated with age of smoking initiation in the UK Biobank (−0.001 log-transformed years (95% confidence interval −0.010 to 0.007)) or in the TAG sample (−0.005 (−0.028 to 0.019); fig 4). Similar results were seen with body fat percentage (−0.007 (−0.023 to 0.010) in UK Biobank and 0.023 (−0.027 to 0.073) in TAG sample; fig 4). 

By contrast, each standard deviation increase in waist circumference was still associated with a younger age of smoking initiation in the UK Biobank (−0.013 log-transformed years (about two months; −0.024 to −0.003), although this relation was not replicated in the TAG sample (−0.020 (−0.048 to 0.008); fig 4). These results indicate that social deprivation is unlikely to explain our observed associations between adiposity parameters and being a smoker and smoking intensity.

### Biological basis for association of body mass index on smoking habits

We further explored the mechanistic basis of the association between the genetic instrument of body mass index and smoking habits by separately analysing SNPs clustering in neuronal pathways. We observed a particularly prominent role for these SNPs with risk of ever being a smoker and smoking intensity (supplementary figs N and P). Using the neuronal-body mass index genetic score, the odds ratio for being an ever smoker was estimated as 1.21 (95% confidence interval 1.14 to 1.28) in UK Biobank and 1.26 (1.08 to 1.47) in the TAG sample. Using the non-neuronal-body mass index genetic score, the corresponding estimated odds ratios were 1.18 (1.13 to 1.23) and 1.12 (0.94 to 1.33), respectively (supplementary fig N; and funnel plots in supplementary fig B). 

Similarly, the association between number of cigarettes smoked per day and neuronal-body mass index genetic score was 1.41 extra cigarettes (95% confidence interval 0.89 to 1.93) in UK Biobank and 1.81 (0.72 to 2.89) in the TAG sample. A weaker or null association was observed using the non-neuronal-body mass index genetic score (0.88 (0.50 to 1.26) and 0.62 (−0.58 to 1.83), respectively; supplementary fig P and funnel plots in supplementary fig H). However, the heterogeneity tests between neuronal and non-neuronal genetic score analyses were not significant (P>0.10).

### Analyses stratified by sex

Analyses stratified by sex did not indicate major differences in the associations between the genetic scores for body mass index, body fat percentage, and waist circumference with smoking parameters (supplementary figs R-U). However, heterogeneity was observed for the association between body fat percentage and smoking cessation. Each standard deviation increase in body fat percentage was associated with a lower odds of smoking cessation in women (0.61 (95% confidence interval 0.48 to 0.76)), but not in men (0.96 (0.78 to 1.18); P_sex-heterogeneity_=0.003; supplementary fig S).

## Discussion

Based on comprehensive genetic data from nearly 450 000 individuals,^27^ our study provides evidence that differences in body mass index and body fat distribution causally influence different aspects of smoking behaviour, including the risk of individuals taking up smoking, smoking intensity, and smoking cessation. These results highlight the role of obesity in influencing smoking initiation and cessation, which could have implications for public health interventions aiming to reduce the prevalence of these important risk factors. 

### Relation between obesity and smoking

Based on our Mendelian randomisation analysis from UK Biobank and the TAG consortium, we observed consistent risk increases of being a smoker with increased increments in body mass index and waist circumference. These associations contrast with those attained by directly measured risk factors, which reflect an inverse association between general adiposity and current smoking status. Previous Mendelian randomisation studies have provided evidence for a causal role of smoking on body weight reduction.^28^ ^29^ These observations highlight a complex bidirectional relation between obesity and tobacco smoking, that:

- Taking up smoking reduces obesity in a causal manner, possibly because of a reduced appetite in smokers.^7^ ^8^
- Higher levels of obesity increase the risk of individuals taking up smoking, as well as smoking intensity.

The fact that smokers generally have a lower body mass index suggests that the effect of smoking on general obesity is stronger than the countering effect of obesity on smoking. In addition, the previously reported correlation between measured waist circumference and smoking status and intensity,^9^ together with the supporting Mendelian randomisation analysis,^30^ suggests that smoking leads to the accumulation of central obesity. Obesity is now recognised as one of the most important health hazards accounting for a large fraction of early deaths worldwide. Given the overwhelmingly detrimental impact of tobacco exposure on health in general^31^ as well as on the risk of multiple cancers and chronic diseases,^32^ our results highlight the importance of jointly considering the risk of both smoking and obesity in any population or in clinical interventions aiming to reduce the prevalence of these risk factors. This joint consideration might be of particular importance for overweight children and young adults who are at risk of taking up smoking. Finally, we observed an inverse association between body fat percentage and smoking cessation in women, but not men. This result could explain some of the causes of unsuccessful attempts to quit smoking, reinforcing the need of combining weight control and smoking cessation strategies, particularly in women.

### Evidence for common neurobiological basis between adiposity and smoking and its implication in clinical and public health interventions

We found that associations of body mass index with measures of tobacco exposure appeared to be primarily driven by SNPs clustering in neuronal pathways. This observation suggests a common biological basis for addictive behaviours, such as nicotine addiction and higher energy intake.^33^ ^34^ This could result in overweight smokers maintaining smoking at higher rates as a result of a genetically predisposed compulsive behaviour.^35^

### Study limitations

As in any Mendelian randomisation analysis, several assumptions were made, including that the genetic instruments were associated with the risk factor of interest, were independent of potential confounders, and could only affect the outcome through the risk factor and not through alternative pathways (that is, through pleiotropy). We note that the first assumption was satisfied because robustly associated gene variants were identified from the largest genome wide association study for each obesity parameter. Whether the other two assumptions held was not readily testable, although we conducted thorough sensitivity analyses that did not highlight any obvious violation of these assumptions. Secondly, a potential confounder of our results was population stratification by sociodemographic factors. Indeed, it was previously shown that the genetic instrument for body mass index was associated with various factors related to social class among women, including lower annual household income and level of deprivation.^36^ However, no such associations were seen in men. In our study, the associations between the genetic instruments of obesity and individuals taking up smoking and smoking intensity were consistently observed in both men and women, separately, and also when we excluded SNPs that were potentially linked to social deprivation. Therefore, apart from the inverse association between body fat percentage and smoking cessation observed in women only, population stratification by sociodemographic factors would not seem likely to explain those results.

**What is already known on this topic**

Smokers have lower body weight on average than non-smokers, but tend to gain weight after quitting smoking; however, active smokers who smoke more intensively tend to weigh more than light smokers
A link between obesity and smoking behaviour could have implications for weight control and smoking prevention strategies, as well as for prevention of multiple non-communicable diseases
The influence of obesity on smoking behaviour is difficult to assess in traditional observational studies

**What this study adds**

Genetic data from about 375 000 participants from the UK Biobank study were analysed, with replication in about 74 000 participants from Tobacco and Genetics consortium
Mendelian randomisation analyses were based on genetic proxies of adiposity measures (body mass index, body fat percentage, and waist circumference); these genetic proxies are less likely to be affected by confounding and are not influenced by reverse causation
Higher levels of general and abdominal adiposity were found to influence smoking status and smoking intensity (number of cigarettes smoked per day)

## Web extra

Extra material supplied by authors

**Web appendix:** Supplementary material

## Notes

Contributors: RC-T, MJ, PB, and RMM initiated and designed this investigation, with important contributions from PCH, CLR, and GDS. RC-T conducted the statistical analysis for this investigation and wrote the first draft of the manuscript, with important contributions from RMM, MJ, PCH, CLR, GDS, and PB. All authors contributed to the interpretation of the study results and critical revision of the manuscript. RC-T and MJ are joint first authors. The corresponding author attests that all listed authors meet authorship criteria and that no others meeting the criteria have been omitted.

Ethical approval: Each participating study obtained informed consent from the study participants and approval from its institutional review board.

Data sharing: No additional data available.

The lead author affirms that the manuscript is an honest, accurate, and transparent account of the study being reported; that no important aspects of the study have been omitted; and that any discrepancies from the study as planned (and, if relevant, registered) have been explained.
