# Supplementary material for: Role of obesity in smoking behaviour: Mendelian randomisation study in UK Biobank
Source: BMJ. 2018 May 16;361:k1767. doi: 10.1136/bmj.k1767 (PMC5953237; doi:10.1136/bmj.k1767)
Supplement: Supplementary file 1 — Web appendix: Supplementary material [file brep040714.ww.pdf]

Web appendix: Supplementary material

Supplementary methods

UK Biobank sample quality control

UK Biobank comprised a total of 487,409 participants with genetic data. Individuals were excluded following these criteria: heterozygosity rates lower or higher than 6 standard deviations (SD) from the mean (419 individuals); genetic sex discrepancy (308 individuals); other genetic background than Caucasian (78,435 individuals); relatedness index higher than 0.25 (1,065); and lack of adiposity or smoking behaviour measures (34,391 individuals). A total of 372,791 participants had valid genetic data and both adiposity and smoking behaviour measures at recruitment.

MR-Egger method

MR-Egger approach performs a weighted linear regression of the SNP-to-smoking estimates (β_{GD}) on the SNP-to-obesity estimates (β_{GP}), allowing the assessment of bias in the initially estimated causal effects of obesity on smoking behaviour in different ways. The main MR-Egger sensitivity test is the analysis of the regression intercept. This test detects an overall directional pleiotropic contribution of weak instrumental SNPs on the estimated causal effect (assuming that any pleiotropic contribution biasing the risk estimation is acting in the same direction). This potential bias can be visualized using a scatter plot of the individual SNP causal estimates (β_{GD}/β_{GP}) against their strength as instruments (β_{GP}/SE_{GD}^2) showing asymmetry of the distribution. These funnel plots were generated using the *ggplot2* R package (R Project). We applied a modified version of MR-Egger regression which adjusts causal effect estimates for violations of the “No Measurement Error (NOME)” assumption, through a simulation extrapolation (SIMEX) method[22]. Additionally, SNPs can be identified as outliers analyzing the studentized residual of the linear MR-Egger model (nominally significant Bonferroni corrected P-value based on a Student’s t-test with degrees of freedom equal to the number of SNPs minus 2).

Weighted median-based approach

The weighted median estimator is the median of a distribution in which the SNP causal estimates (β_{GD}/β_{GP}) have been ordered and represent percentiles of this distribution[23], which is less sensitive to the effect of pleiotropic variants behaving as outliers.

MR modal-based approach

The modal-based estimate is the mode of the SNP causal estimates (β_{GD}/β_{GP}) distribution, and its consistency with the true causal effect relies on the assumption termed the ZEro Modal Pleiotropy Assumption (ZEMPA)[24]. This assumption implies the most common biasing effect from SNP ratio estimates is equal to 0. Therefore, the largest number of similar SNP ratio estimates comes from valid instruments, even if the majority of instruments are invalid.

MR likelihood-based approach

We used a likelihood-based approach[27] to undertake MR based on summary data. This method is considered the most accurate to estimate causal effects when there is a continuous linear or log-linear association between the exposure factor and the outcome. This approach provides similar association estimates than genetic scores at individual level data because of the similar sensitivity of both methods towards outliers or strong influencing SNPs, which is different than the MR-Egger approach.

Sex interaction

As the selected BMI, body fat percentage and waist circumference genetic variants have similar effects in men and women[15-17], we used the same genetic instruments for both sex categories. We investigated the between-sex heterogeneity of causal effects by estimating the percentage of variance that is attributable to sex heterogeneity (I^2 statistic), and the P value derived from Q statistic for heterogeneity ($P_{Heterogeneity}$), assuming a fixed-effect model of 1 degree of freedom.

Supplementary figures

Supplementary figure A- Power estimation of risk detection per one standard deviation (SD) increase in body-mass index (BMI), waist circumference (WC) and body fat percentage (BF%) reflected by our genetic instruments in both UK Biobank (UKB) and Tobacco and Genetics (TAG) Consortium samples.

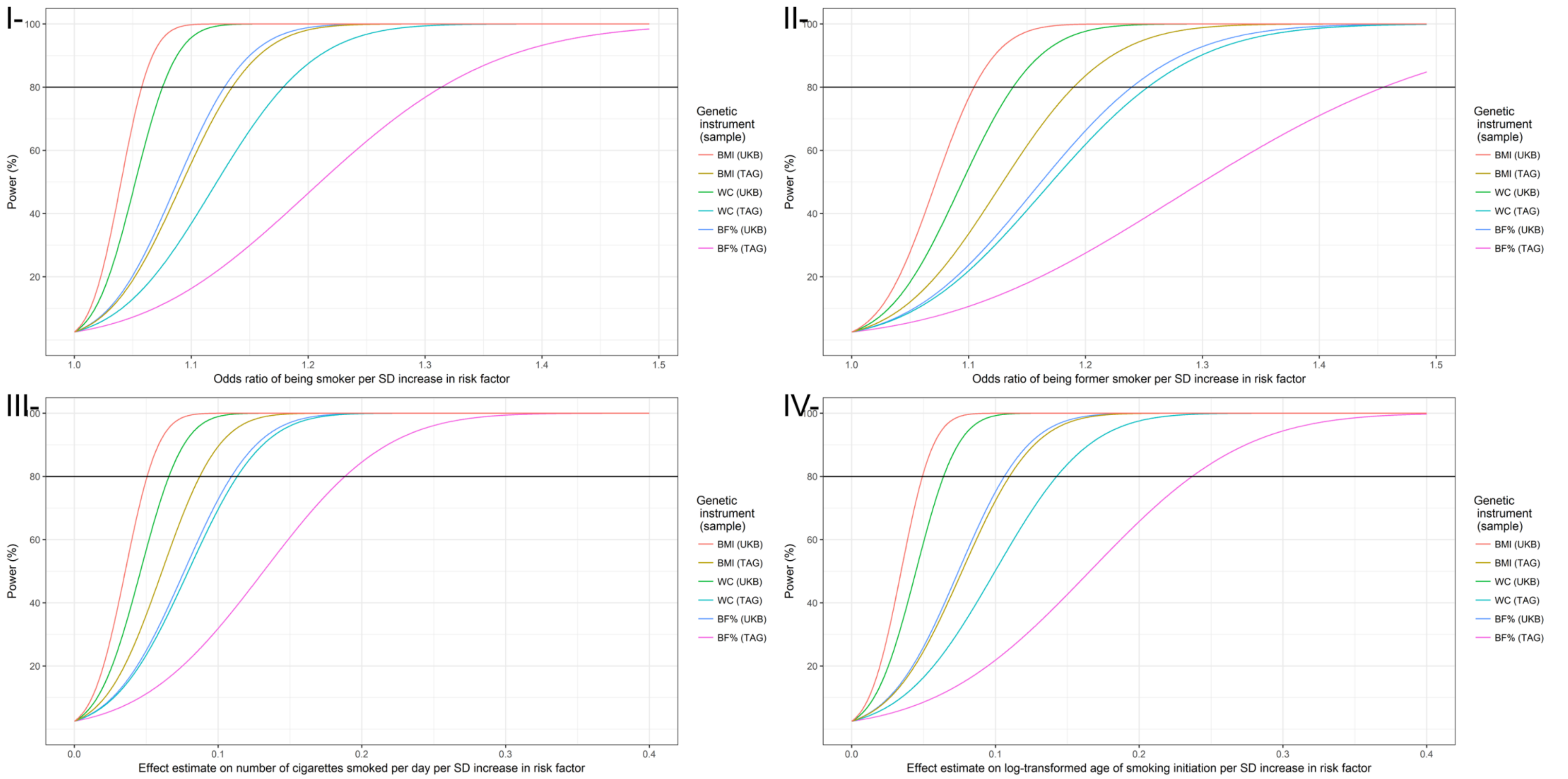

Supplementary figure B- Sensitivity analyses for associations between genetically determined 1 standard deviation increase of BMI and smoking status (ever vs never smokers). The histograms reflect the distribution of risk estimates from the “leave-one out” analysis applied to each MR method. Individual SNPs that strongly influence the overall effect estimate will be reflected with a deviating effect estimate.

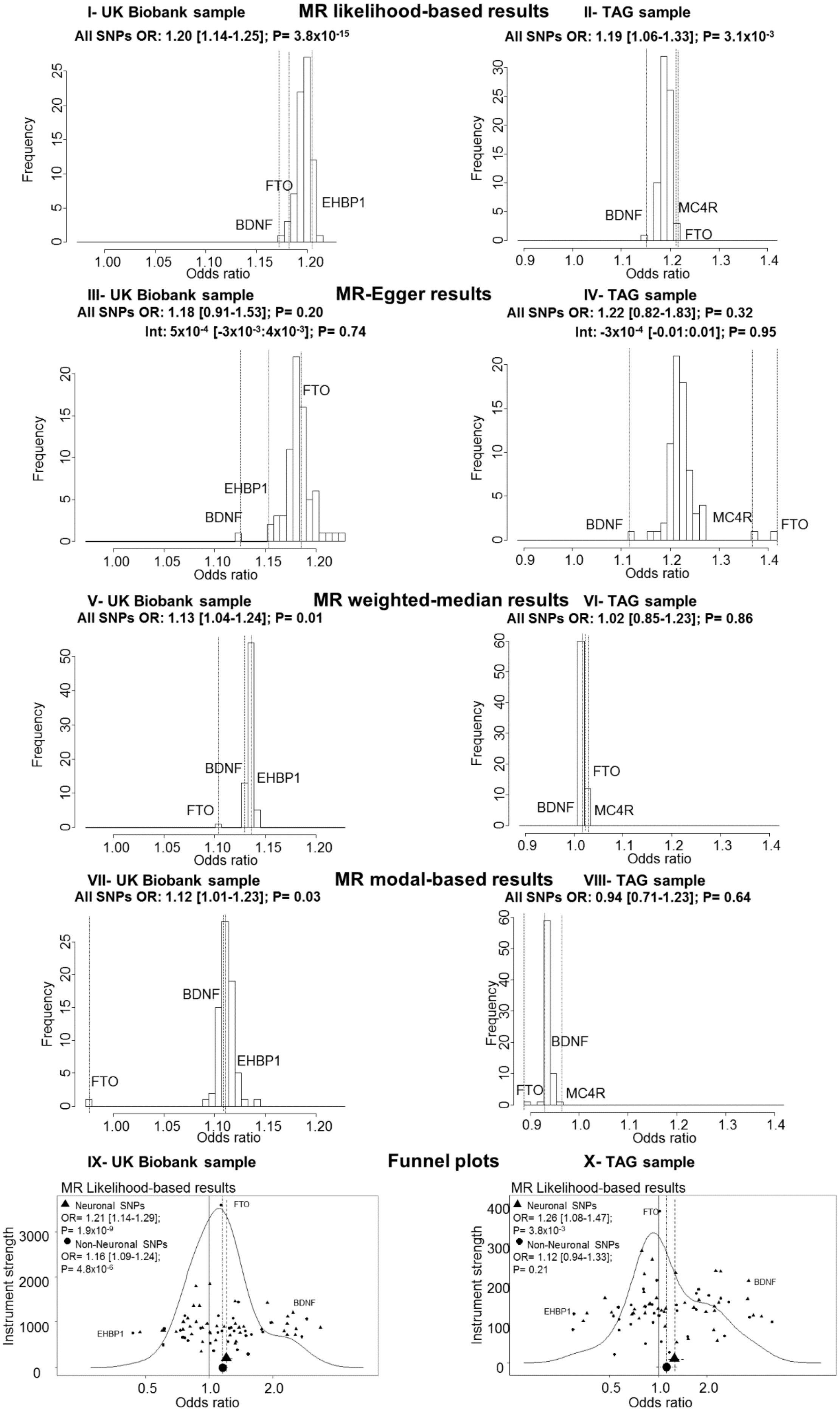

Supplementary figure C- Sensitivity analyses for associations between genetically determined 1 standard deviation increase of body fat percentage and smoking status (ever vs never smokers). The histograms reflect the distribution of risk estimates from the “leave-one out” analysis applied to each MR method. Individual SNPs that strongly influence the overall effect estimate will be reflected with a deviating effect estimate.

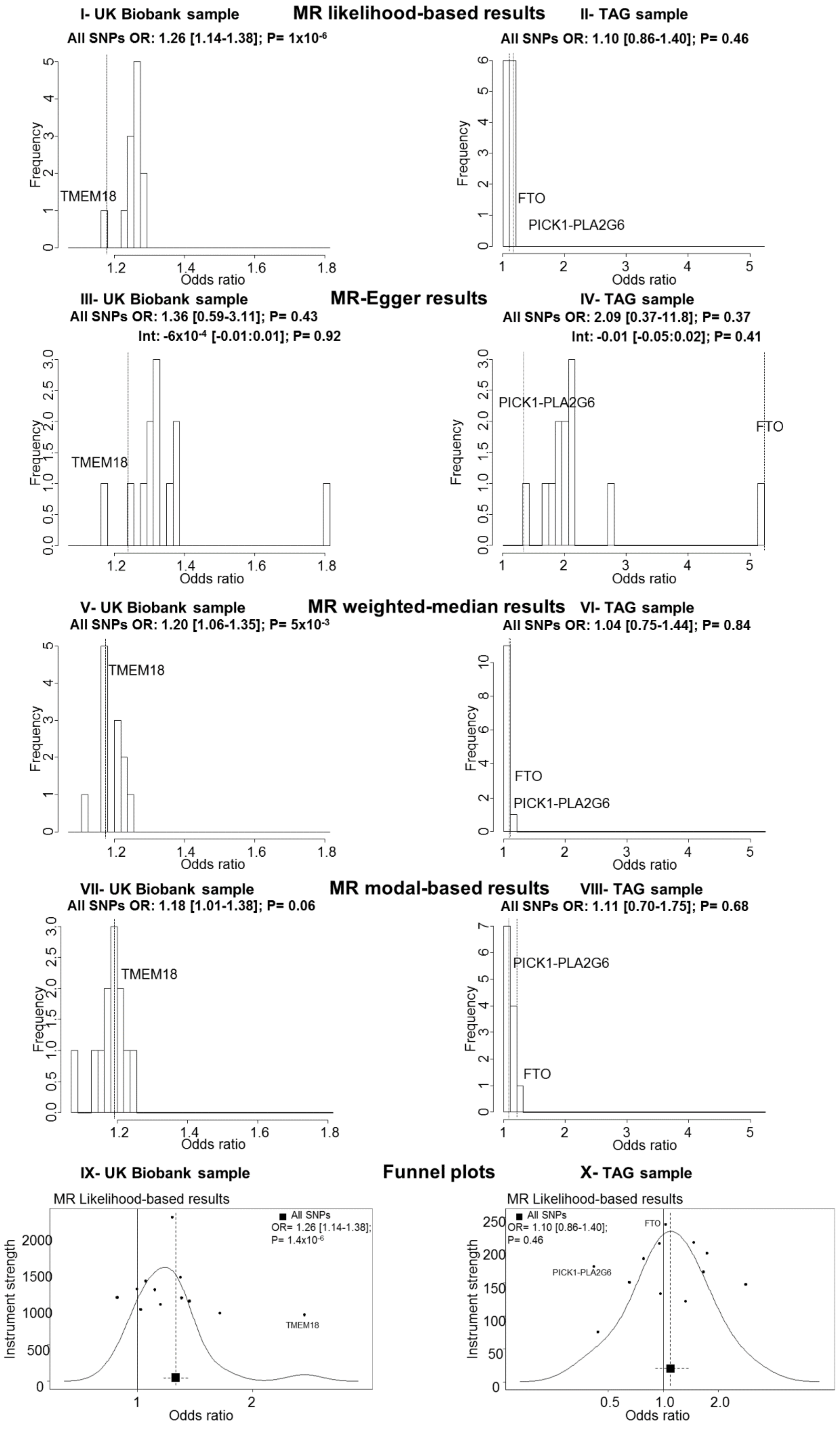

Supplementary figure D- Sensitivity analyses for associations between genetically determined 1 standard deviation increase of waist circumference and smoking status (ever vs never smokers). The histograms reflect the distribution of risk estimates from the “leave-one out” analysis applied to each MR method. Individual SNPs that strongly influence the overall effect estimate will be reflected with a deviating effect estimate.

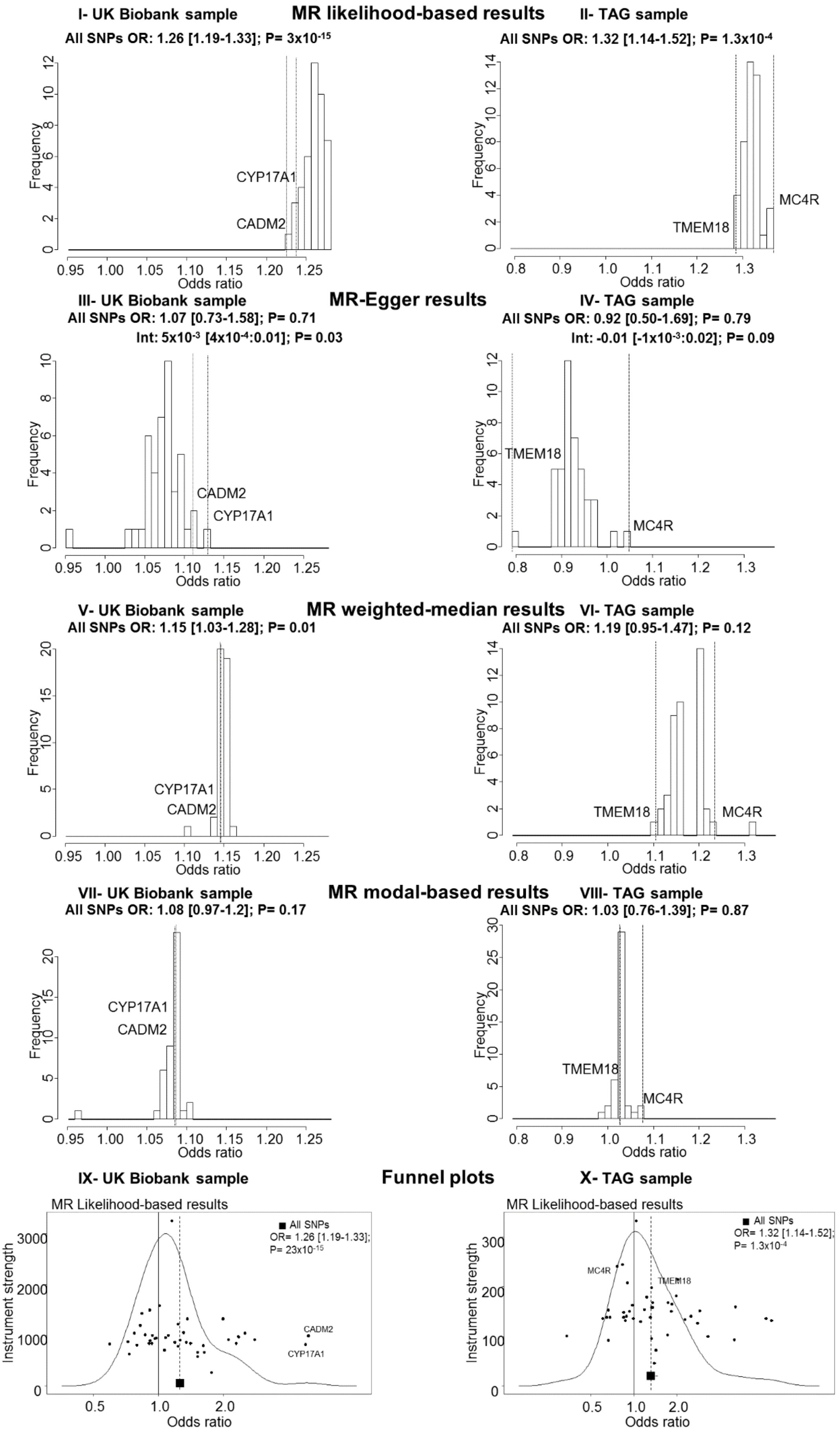

Supplementary figure E- Sensitivity analyses for associations between genetically determined 1 standard deviation increase of BMI and smoking cessation (former vs current smokers). The histograms reflect the distribution of risk estimates from the “leave-one out” analysis applied to each MR method. Individual SNPs that strongly influence the overall effect estimate will be reflected with a deviating effect estimate.

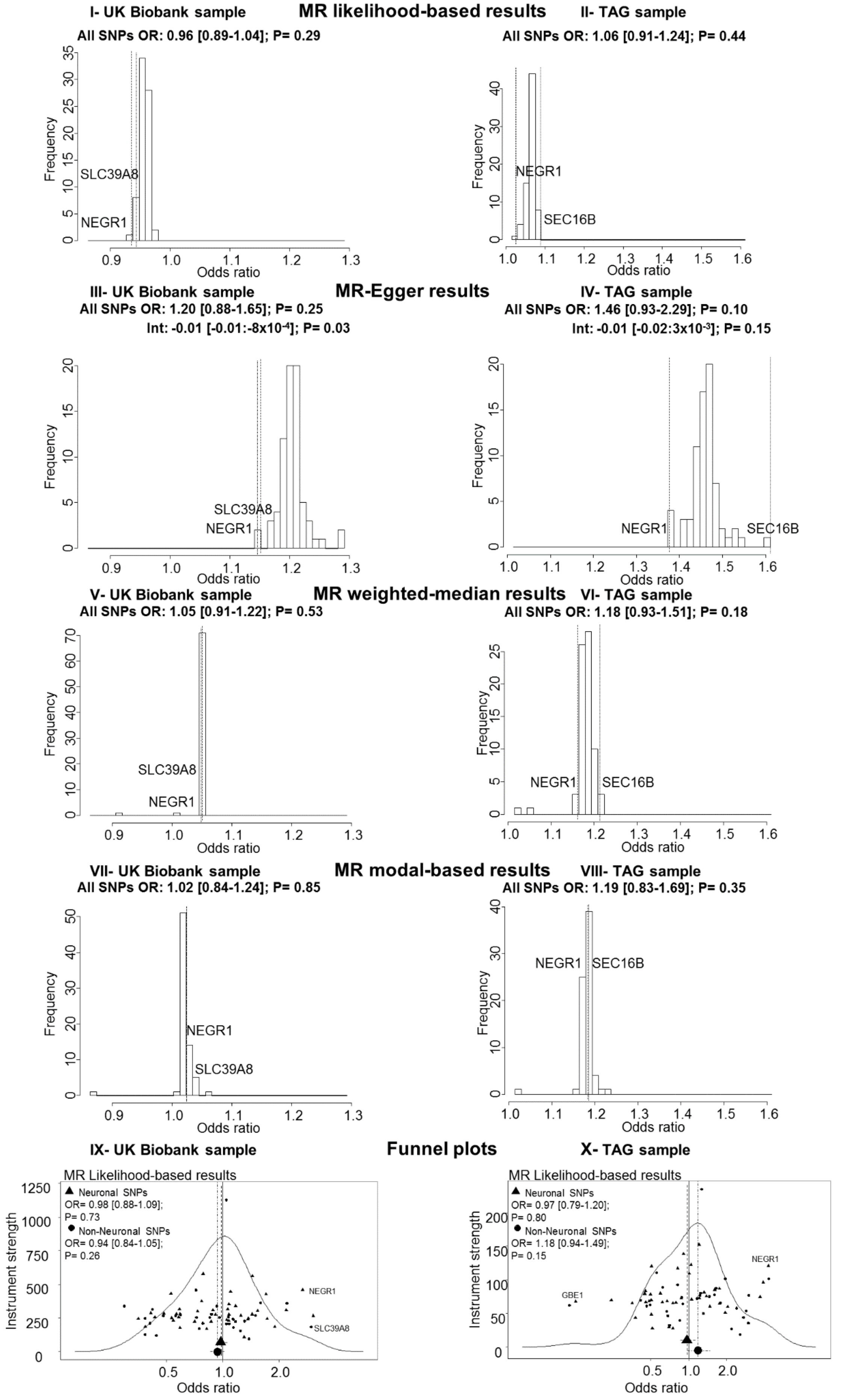

Supplementary figure F- Sensitivity analyses for associations between genetically determined 1 standard deviation increase of body fat percentage and smoking cessation (former vs current smokers). The histograms reflect the distribution of risk estimates from the "leave-one out" analysis applied to each MR method. Individual SNPs that strongly influence the overall effect estimate will be reflected with a deviating effect estimate.

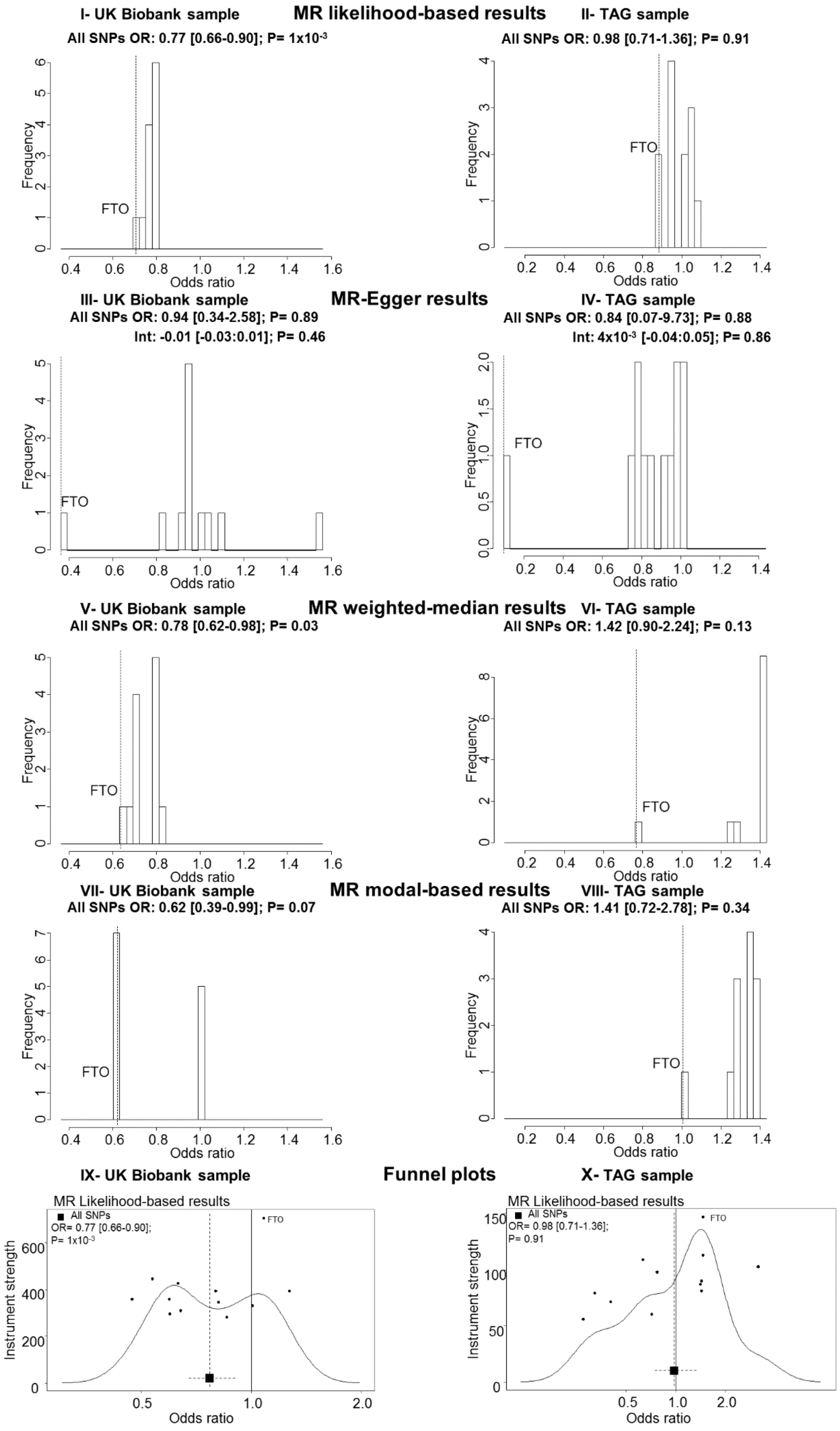

Supplementary figure G- Sensitivity analyses for associations between genetically determined 1 standard deviation increase of waist circumference and smoking cessation (former vs current smokers). The histograms reflect the distribution of risk estimates from the “leave-one out” analysis applied to each MR method. Individual SNPs that strongly influence the overall effect estimate will be reflected with a deviating effect estimate.

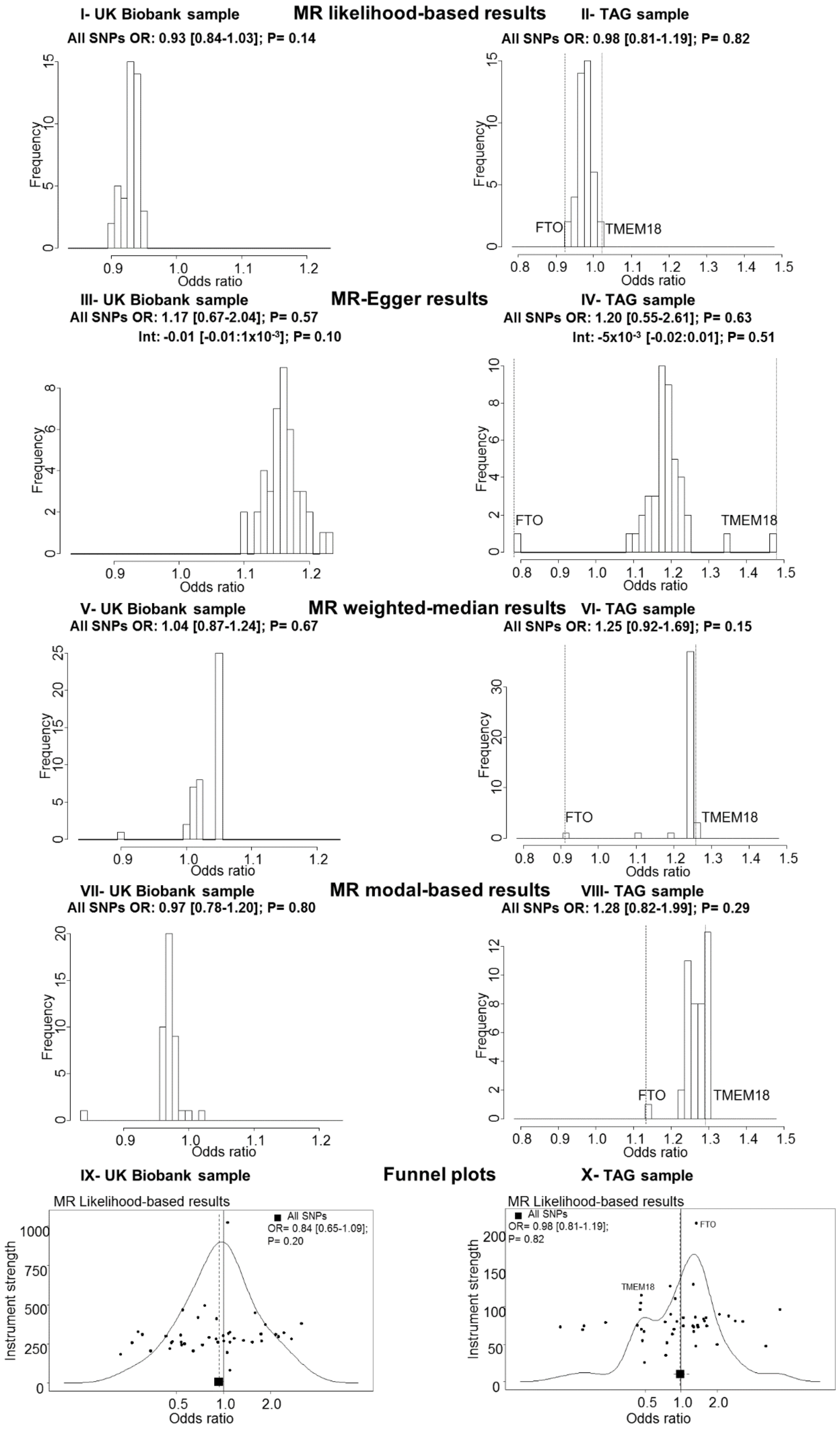

Supplementary figure H- Sensitivity analyses for associations between genetically determined 1 standard deviation increase of BMI and number of cigarettes smoked per day. The histograms reflect the distribution of risk estimates from the “leave-one out” analysis applied to each MR method. Individual SNPs that strongly influence the overall effect estimate will be reflected with a deviating effect estimate.

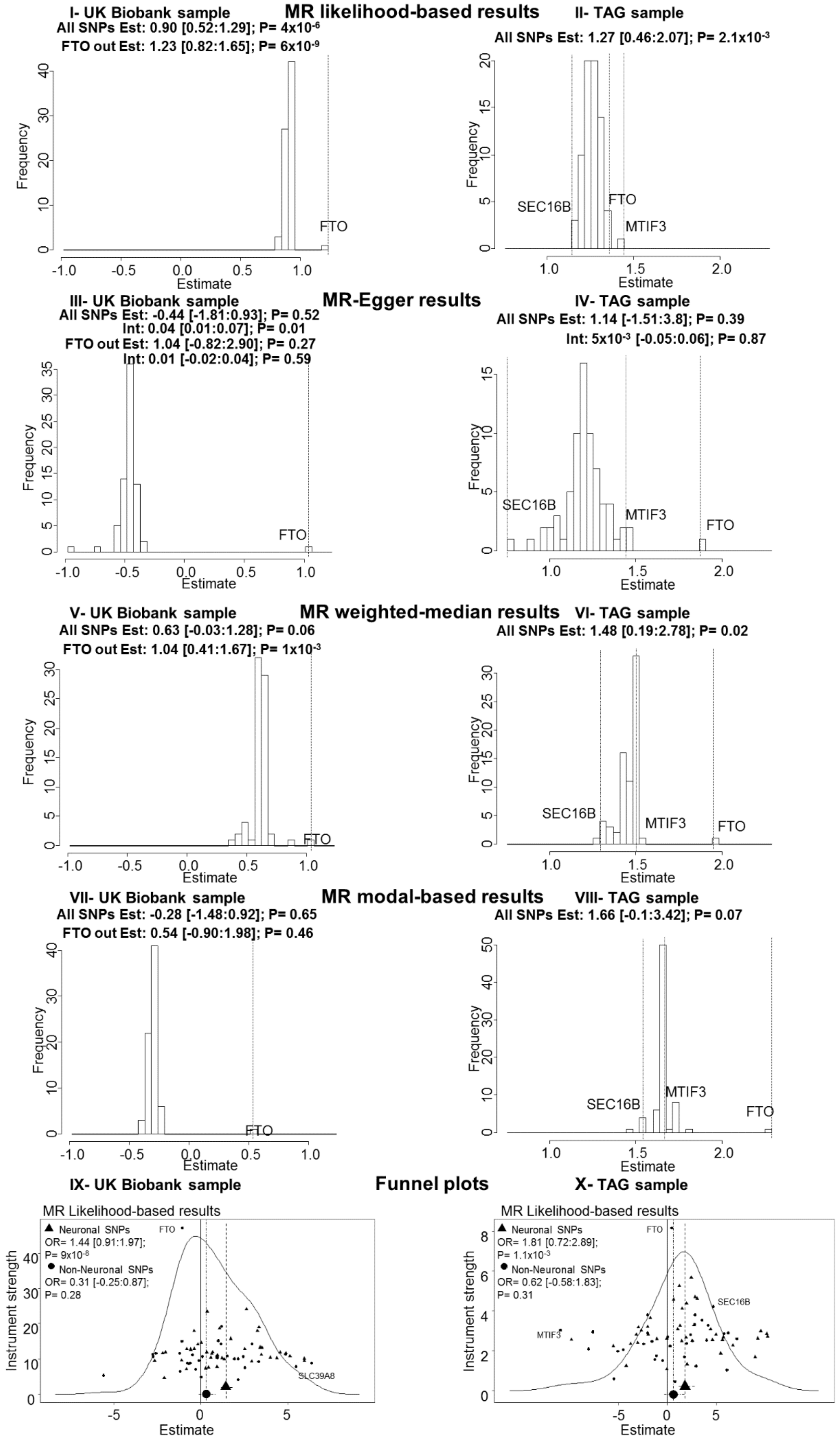

Supplementary figure I- Sensitivity analyses for associations between genetically determined 1 standard deviation increase of body fat percentage and number of cigarettes smoked per day. The histograms reflect the distribution of risk estimates from the "leave-one out" analysis applied to each MR method. Individual SNPs that strongly influence the overall effect estimate will be reflected with a deviating effect estimate.

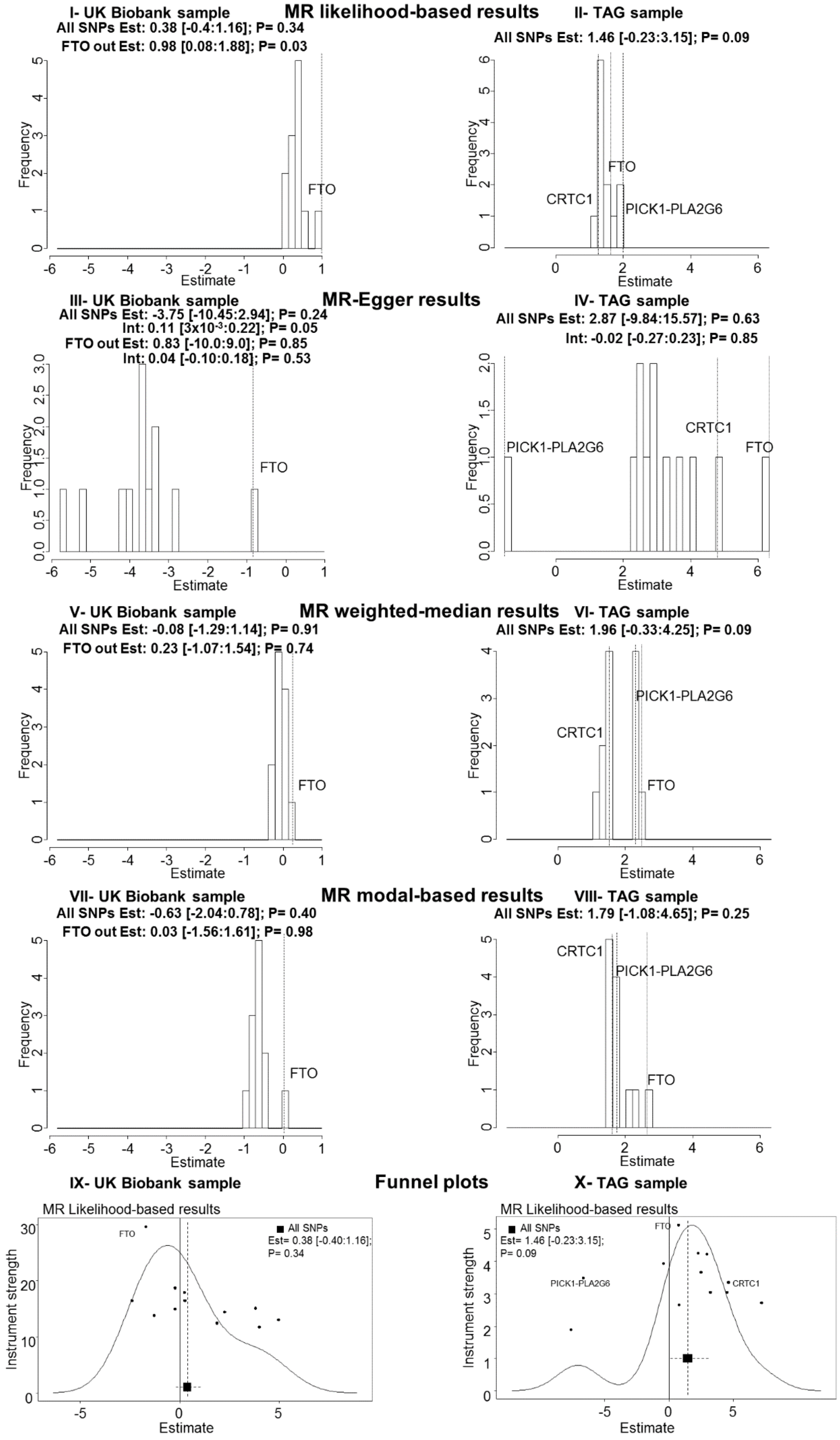

Supplementary figure J- Sensitivity analyses for associations between genetically determined 1 standard deviation increase of waist circumference and number of cigarettes smoked per day. The histograms reflect the distribution of risk estimates from the "leave-one out" analysis applied to each MR method. Individual SNPs that strongly influence the overall effect estimate will be reflected with a deviating effect estimate.

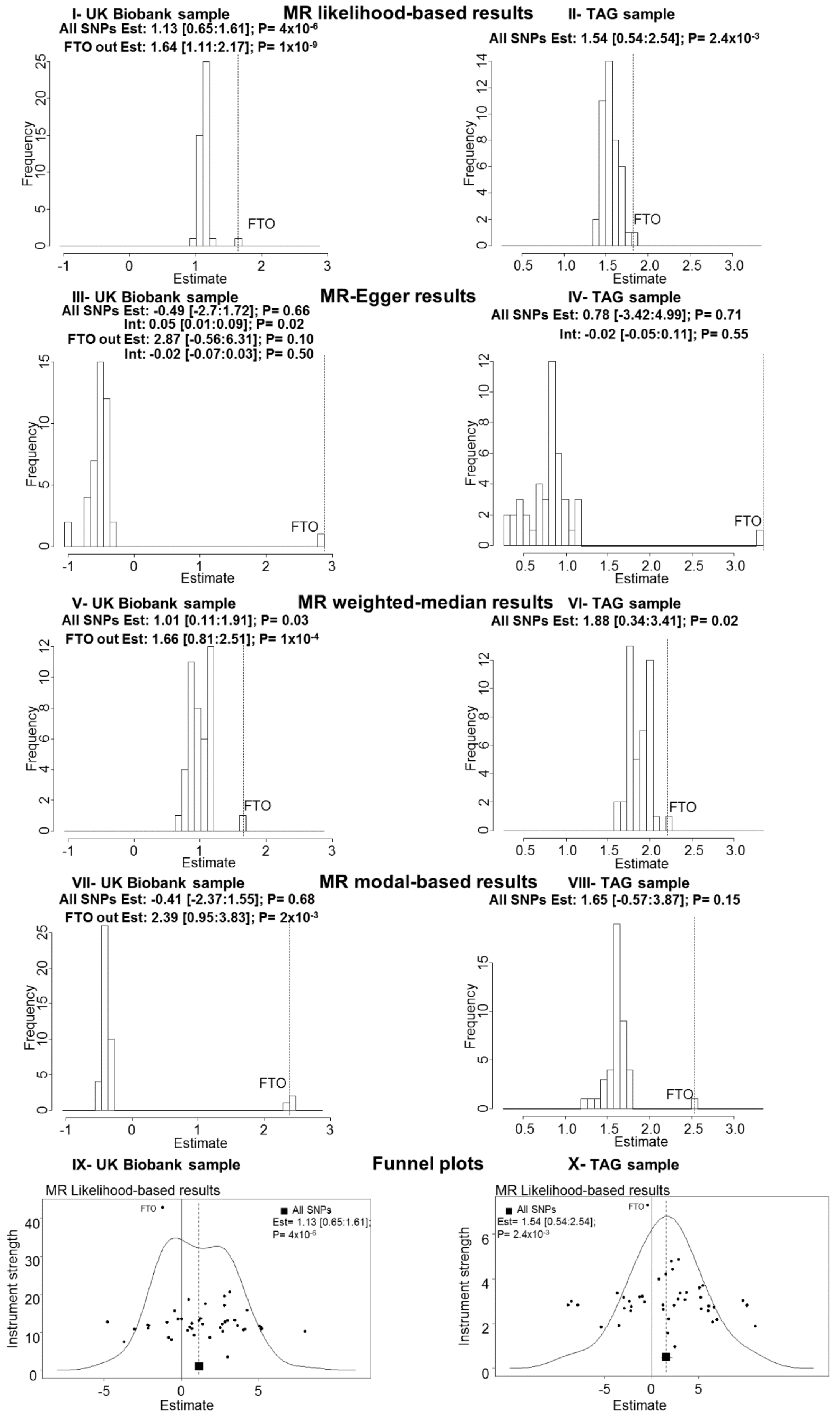

Supplementary figure K- Sensitivity analyses for associations between genetically determined 1 standard deviation increase of BMI and years of age of smoking initiation (log-transformed). The histograms reflect the distribution of risk estimates from the "leave-one out" analysis applied to each MR method. Individual SNPs that strongly influence the overall effect estimate will be reflected with a deviating effect estimate.

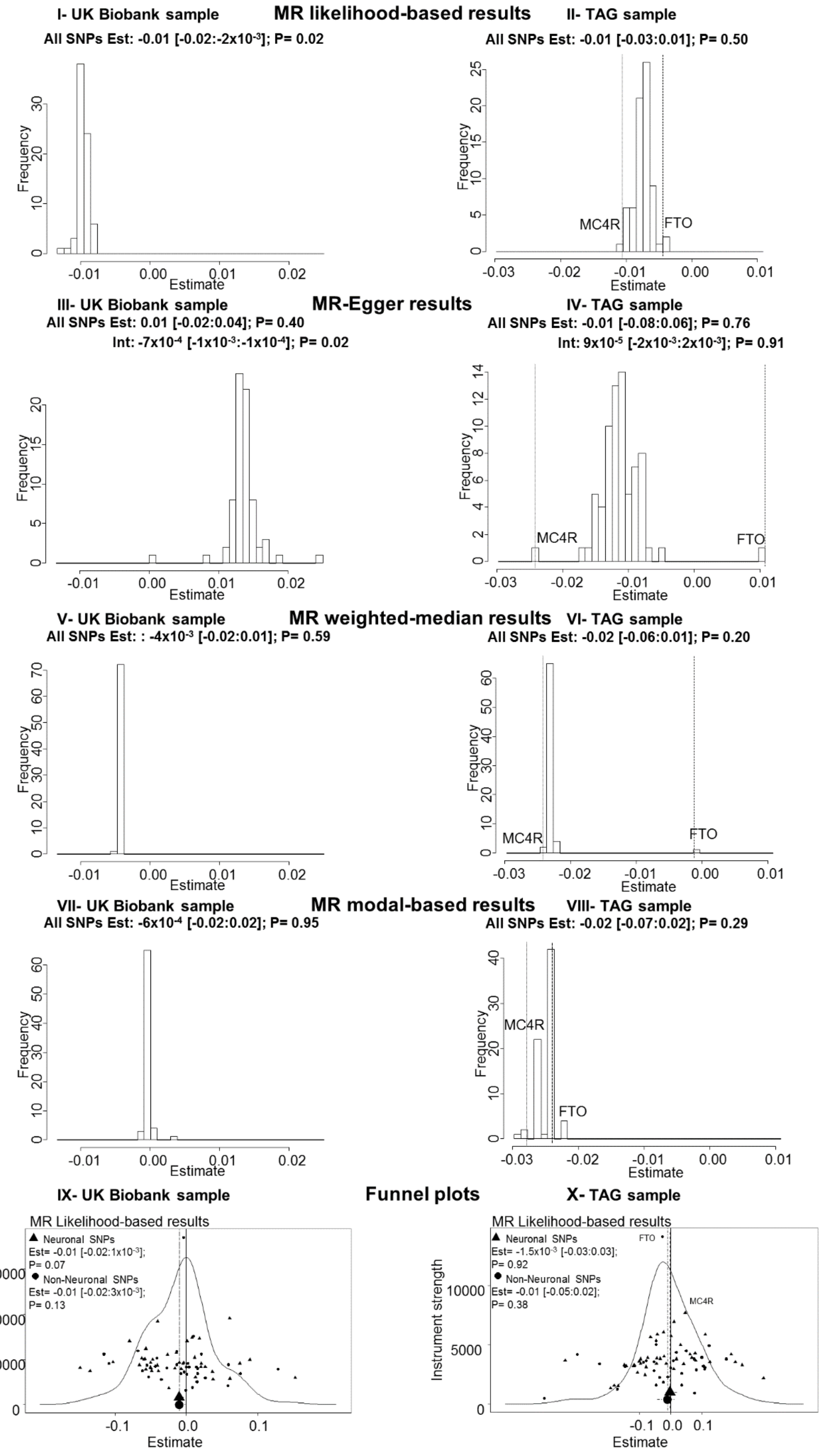

Supplementary figure L- Sensitivity analyses for associations between genetically determined 1 standard deviation increase of body fat percentage and years of age of smoking initiation (log-transformed). The histograms reflect the distribution of risk estimates from the "leave-one out" analysis applied to each MR method. Individual SNPs that strongly influence the overall effect estimate will be reflected with a deviating effect estimate.

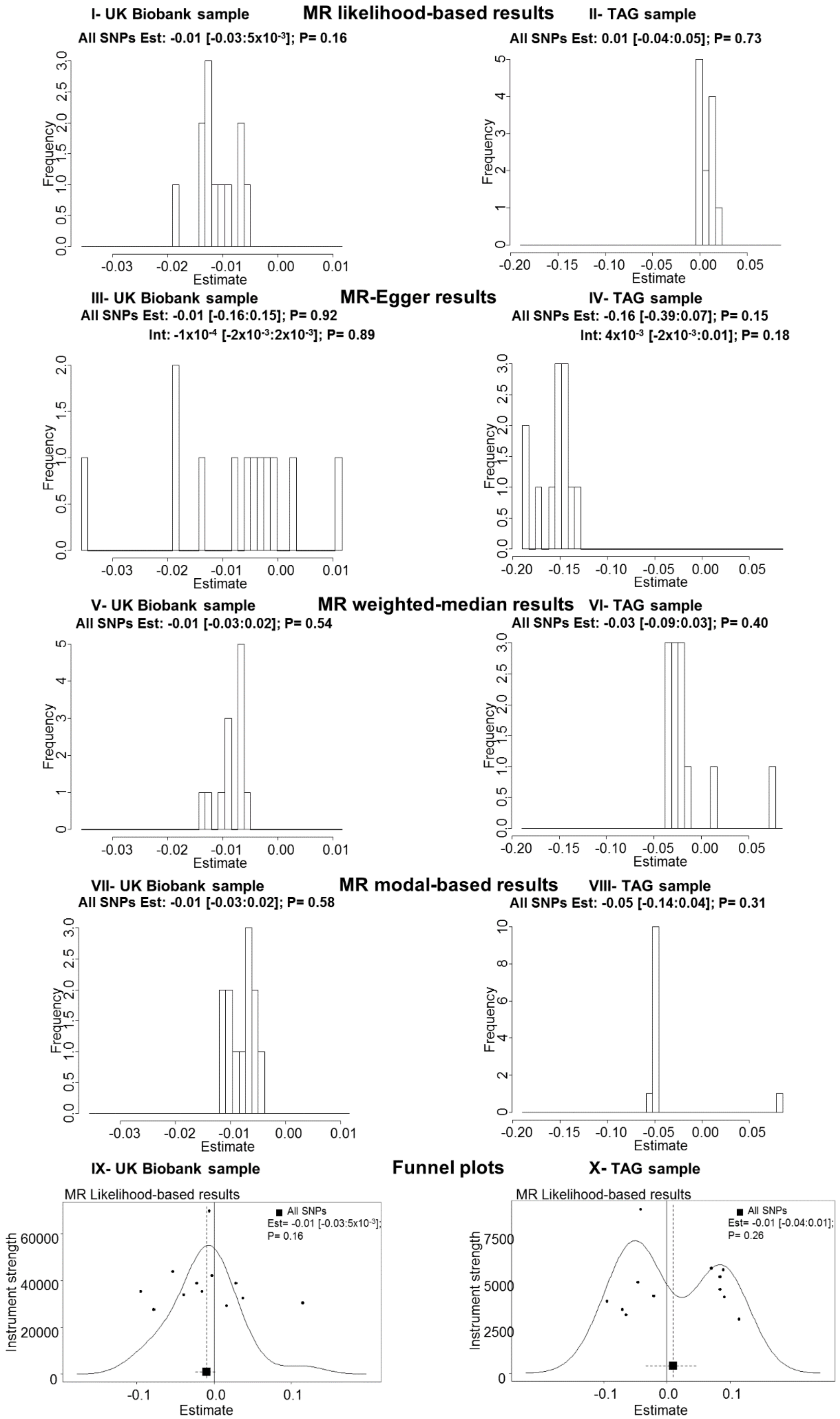

Supplementary figure M- Sensitivity analyses for associations between genetically determined 1 standard deviation increase of waist circumference and years of age of smoking initiation (log-transformed). The histograms reflect the distribution of risk estimates from the "leave-one out" analysis applied to each MR method. Individual SNPs that strongly influence the overall effect estimate will be reflected with a deviating effect estimate.

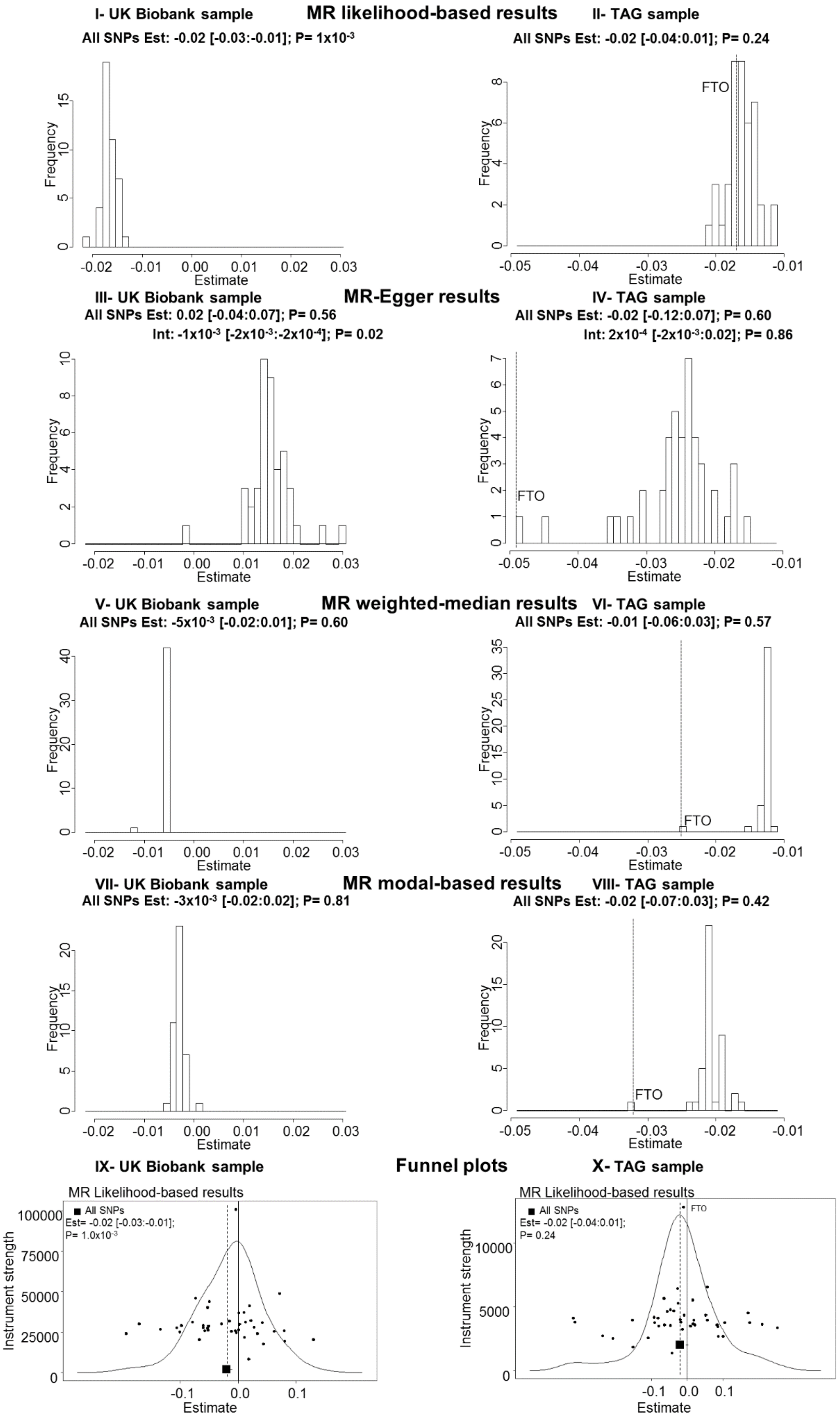

Supplementary figure N- Association between subgroups of adiposity SNPs and smoking status (ever vs never smokers). CI: Confidence interval. P: P value.

Adiposity parameter	Sample	Smoking group	Odds ratio of being smoker	95% CI	P
BMI-Neuronal	UK Biobank	Current smokers	1.24	1.12 to 1.37	$3.6x10^{-5}$
		Ever smokers	1.21	1.14 to 1.28	$8.5x10^{-10}$
	TAG	Ever smokers	1.26	1.08 to 1.47	$3.8x10^{-3}$
BMI-Non-Neuronal	UK Biobank	Current smokers	1.24	1.15 to 1.33	$1.9x10^{-8}$
		Ever smokers	1.18	1.13 to 1.23	$3.2x10^{-14}$
	TAG	Ever smokers	1.12	0.94 to 1.33	0.21
BMI-Non-Deprivation	UK Biobank	Current smokers	1.11	1.02 to 1.21	0.01
		Ever smokers	1.11	1.06 to 1.17	$2.1x10^{-5}$
	TAG	Ever smokers	1.17	1.03 to 1.32	0.02
Body fat percentage-Non-Deprivation	UK Biobank	Current smokers	1.46	1.25 to 1.71	$1.9x10^{-6}$
		Ever smokers	1.24	1.13 to 1.35	$5.6x10^{-6}$
	TAG	Ever smokers	1.05	0.79 to 1.38	0.75
Waist circumference-Non-Deprivation	UK Biobank	Current smokers	1.22	1.10 to 1.35	$1.4x10^{-4}$
		Ever smokers	1.22	1.15 to 1.29	$6.3x10^{-11}$
	TAG	Ever smokers	1.34	1.16 to 1.56	$1.1x10^{-4}$

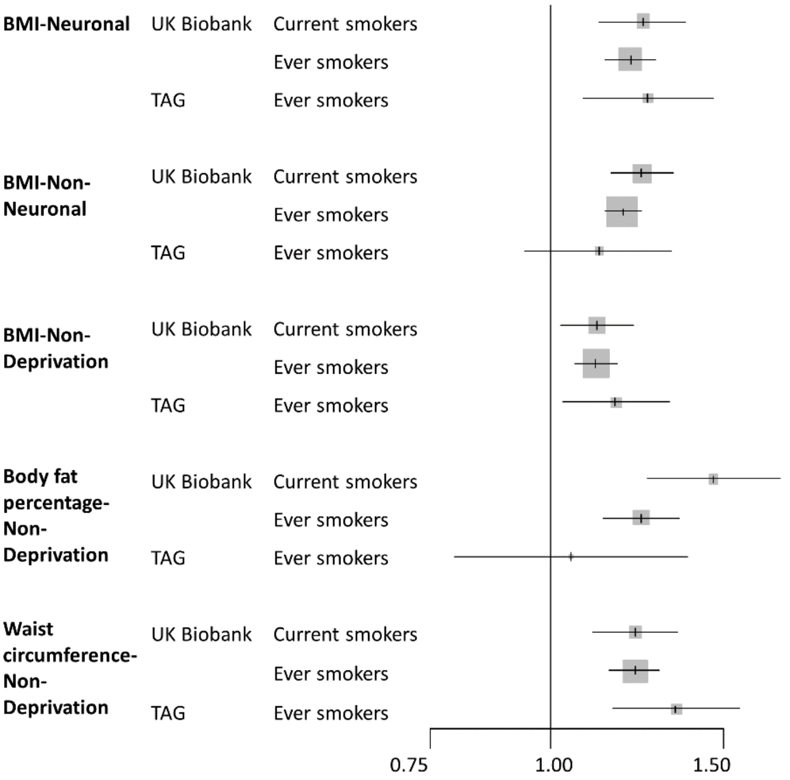

Supplementary figure O- Association between subgroups of adiposity SNPs and smoking cessation (former vs current smokers). CI: Confidence interval. P: P value.

Adiposity parameter	Sample	Odds ratio of being former smoker	95% CI	P
**BMI-Neuronal**	UK Biobank	0.98	0.88 to 1.09	0.73
	TAG	0.97	0.79 to 1.20	0.80
**BMI-Non-Neuronal**	UK Biobank	0.96	0.89 to 1.04	0.29
	TAG	1.18	0.94 to 1.49	0.15
**BMI-Non-Deprivation**	UK Biobank	1.01	0.93 to 1.10	0.84
	TAG	1.13	0.95 to 1.33	0.16
**BF-Non-Deprivation**	UK Biobank	0.81	0.69 to 0.96	0.01
	TAG	1.06	0.73 to 1.53	0.75
**WC-Non-Deprivation**	UK Biobank	1.02	0.92 to 1.13	0.73
	TAG	1.02	0.83 to 1.25	0.85

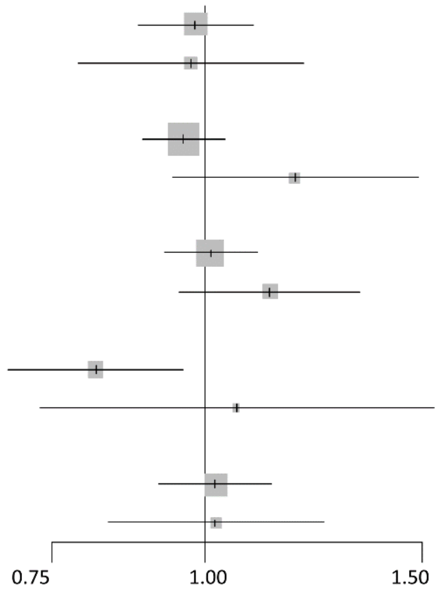

Supplementary figure P- Association between subgroups of adiposity SNPs and number of cigarettes smoked per day. CI: Confidence interval. P: P value.

Adiposity parameter	Sample	Smoking group	Number of extra cigarettes smoked per day	95% CI	P
**BMI-Neuronal**	UK Biobank	Current smokers	1.39	0.48 to 2.30	$2.9x10^{-3}$
		Ever smokers	1.41	0.89 to 1.93	$1.2x10^{-7}$
	TAG	Ever smokers	1.81	0.72 to 2.89	$1.1x10^{-3}$
**BMI-Non-Neuronal**	UK Biobank	Current smokers	1.14	0.48 to 1.80	$7.4x10^{-4}$
		Ever smokers	0.88	0.50 to 1.26	$5.1x10^{-6}$
	TAG	Ever smokers	0.62	-0.58 to 1.83	0.31
**BMI-Non-Deprivation**	UK Biobank	Current smokers	0.75	0.01 to 1.50	0.05
		Ever smokers	0.74	0.31 to 1.16	$6.7x10^{-4}$
	TAG	Ever smokers	1.43	0.55 to 2.30	$1.3x10^{-3}$
**Body fat percentage-Non-Deprivation**	UK Biobank	Current smokers	0.85	-0.54 to 2.25	0.23
		Ever smokers	0.03	-0.76 to 0.83	0.94
	TAG	Ever smokers	0.82	-1.09 to 2.73	0.40
**Waist circumference-Non-Deprivation**	UK Biobank	Current smokers	0.55	-0.36 to 1.45	0.24
		Ever smokers	1.01	0.49 to 1.53	$1.3x10^{-4}$
	TAG	Ever smokers	2.02	0.98 to 3.07	$1.5x10^{-4}$

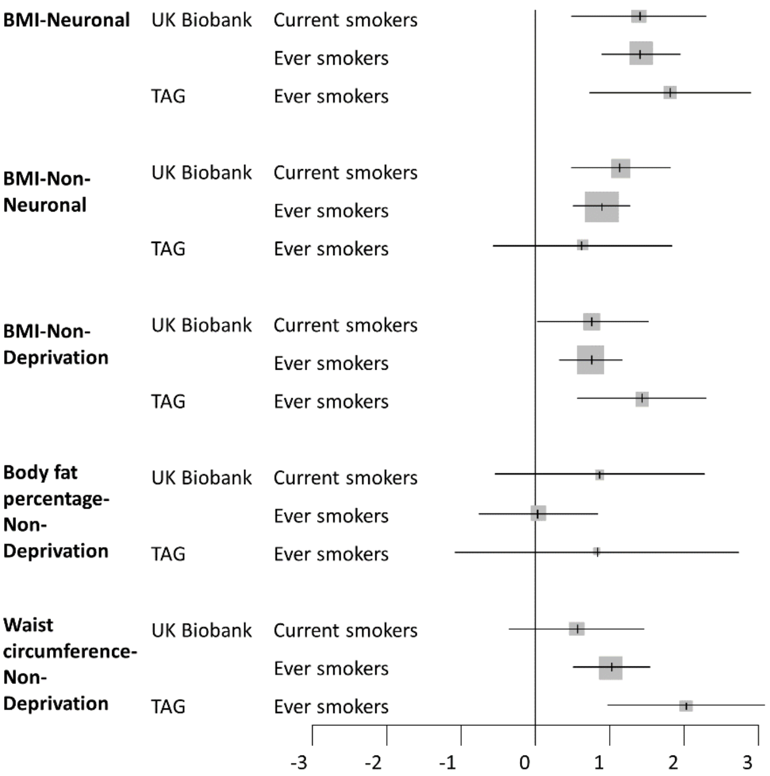

Supplementary figure Q- Association between subgroups of adiposity SNPs and years of age of smoking initiation (log-transformed). CI: Confidence interval. P: P value.

Adiposity parameter	Sample	Years of age of smoking initiation (log-transformed)	95% CI	P
BMI-Neuronal	UK Biobank	-0.01	-0.02 to 6.1×10^{-4}	0.06
	TAG	0.00	-0.03 to 0.03	0.92
BMI-Non-Neuronal	UK Biobank	-0.01	-0.02 to -1.8×10^{-3}	0.02
	TAG	-0.01	-0.05 to 0.02	0.38
BMI-Non-Deprivation	UK Biobank	-1.4×10^{-3}	-0.01 to 0.01	0.75
	TAG	-4.7×10^{-3}	-0.03 to 0.02	0.70
BF-Non-Deprivation	UK Biobank	-0.01	-0.02 to 0.01	0.41
	TAG	0.02	-0.03 to 0.07	0.37
WC-Non-Deprivation	UK Biobank	-0.01	-0.02 to -2.7×10^{-3}	0.01
	TAG	-0.02	-0.05 to 0.01	0.17

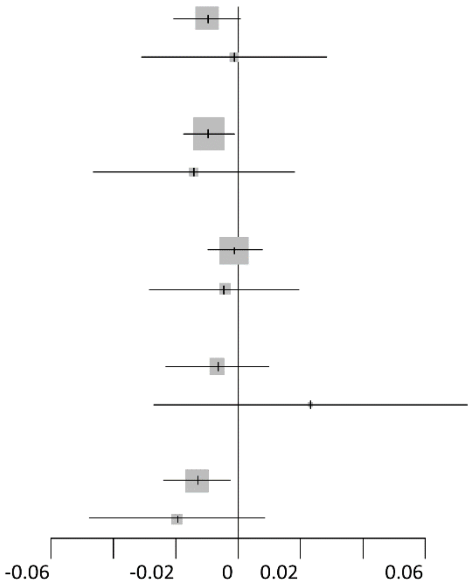

Supplementary figure R- Association between genetically determined 1 standard deviation of adiposity parameters and smoking status (ever vs never smokers) in UK Biobank stratified by sex. CI: Confidence interval. P: P value.

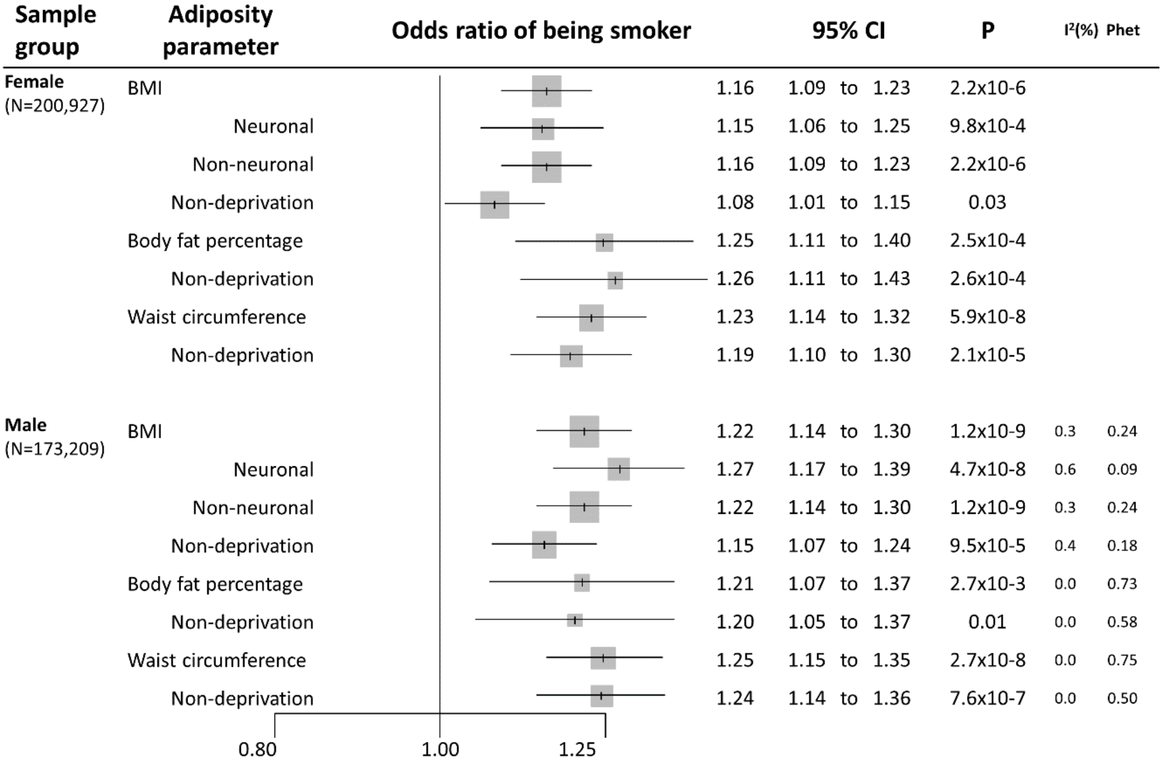

Sample group	Adiposity parameter	Odds ratio of being smoker	95% CI	P	I^2(%)	Phet
**Female** (N=200,927)	BMI	1.16	1.09 to 1.23	2.2x10-6		
	Neuronal	1.15	1.06 to 1.25	9.8x10-4		
	Non-neuronal	1.16	1.09 to 1.23	2.2x10-6		
	Non-deprivation	1.08	1.01 to 1.15	0.03		
	Body fat percentage	1.25	1.11 to 1.40	2.5x10-4		
	Non-deprivation	1.26	1.11 to 1.43	2.6x10-4		
	Waist circumference	1.23	1.14 to 1.32	5.9x10-8		
	Non-deprivation	1.19	1.10 to 1.30	2.1x10-5		
**Male** (N=173,209)	BMI	1.22	1.14 to 1.30	1.2x10-9	0.3	0.24
	Neuronal	1.27	1.17 to 1.39	4.7x10-8	0.6	0.09
	Non-neuronal	1.22	1.14 to 1.30	1.2x10-9	0.3	0.24
	Non-deprivation	1.15	1.07 to 1.24	9.5x10-5	0.4	0.18
	Body fat percentage	1.21	1.07 to 1.37	2.7x10-3	0.0	0.73
	Non-deprivation	1.20	1.05 to 1.37	0.01	0.0	0.58
	Waist circumference	1.25	1.15 to 1.35	2.7x10-8	0.0	0.75
	Non-deprivation	1.24	1.14 to 1.36	7.6x10-7	0.0	0.50

Supplementary figure S- Association between genetically determined 1 standard deviation of adiposity parameters and smoking cessation (former vs current smokers) in UK Biobank stratified by sex. CI: Confidence interval. P: P value.

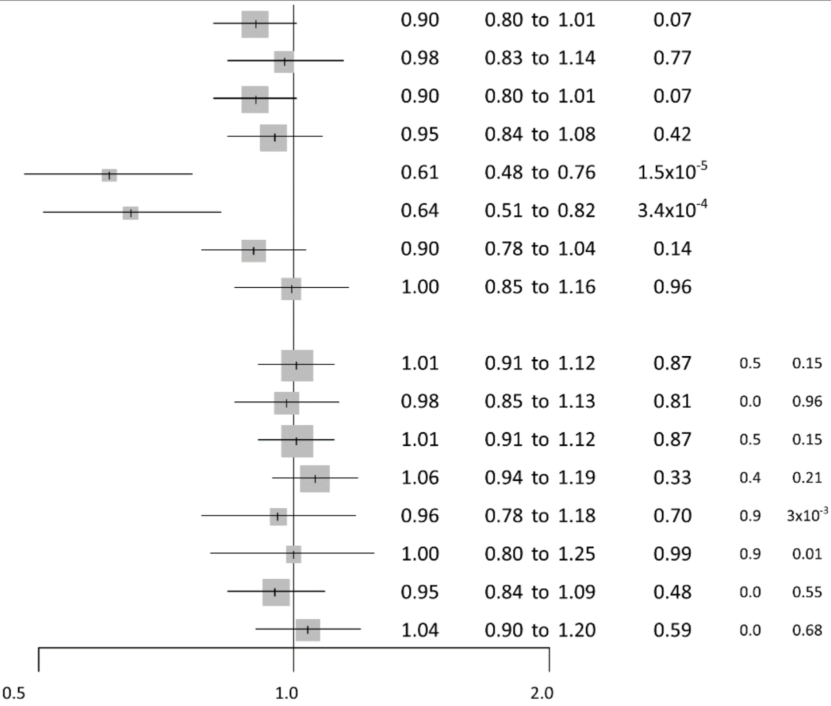

Sample group	Adiposity parameter	Odds ratio of being former smoker	95% CI	P	I^2(%)	Phet
Female (N=81,404)	BMI	0.90	0.80 to 1.01	0.07		
	Neuronal	0.98	0.83 to 1.14	0.77		
	Non-neuronal	0.90	0.80 to 1.01	0.07		
	Non-deprivation	0.95	0.84 to 1.08	0.42		
	BF%	0.61	0.48 to 0.76	$1.5x10^{-5}$		
	Non-deprivation	0.64	0.51 to 0.82	$3.4x10^{-4}$		
	WC	0.90	0.78 to 1.04	0.14		
	Non-deprivation	1.00	0.85 to 1.16	0.96		
Male (N=88,348)	BMI	1.01	0.91 to 1.12	0.87	0.5	0.15
	Neuronal	0.98	0.85 to 1.13	0.81	0.0	0.96
	Non-neuronal	1.01	0.91 to 1.12	0.87	0.5	0.15
	Non-deprivation	1.06	0.94 to 1.19	0.33	0.4	0.21
	BF%	0.96	0.78 to 1.18	0.70	0.9	$3x10^{-3}$
	Non-deprivation	1.00	0.80 to 1.25	0.99	0.9	0.01
	WC	0.95	0.84 to 1.09	0.48	0.0	0.55
	Non-deprivation	1.04	0.90 to 1.20	0.59	0.0	0.68

Supplementary figure T- Association between genetically determined 1 standard deviation of adiposity parameters and number of cigarettes smoked per day in UK Biobank ever smokers stratified by sex. CI: Confidence interval. P: P value.

Sample group	Adiposity parameter	Number of extra cigarretes smoked per day		95% CI	P	I^2(%)	Phet
Female (N=54,229)	BMI		0.84	0.39 to 1.30	$2.5x10^{-4}$		
	Neuronal		1.43	0.81 to 2.06	$6.7x10^{-6}$		
	Non-neuronal		0.84	0.39 to 1.30	$2.5x10^{-4}$		
	Non-deprivation		0.73	0.22 to 1.24	$4.9x10^{-3}$		
	Body fat percentage		0.37	-0.52 to 1.27	0.41		
	Non-deprivation		$-4x10^{-4}$	-0.95 to 0.95	1.00		
	Waist circumference		0.85	0.29 to 1.40	3.0x10-3		
	Non-deprivation		0.64	0.02 to 1.26	0.04		
Male (N=59,066)	BMI		0.92	0.32 to 1.51	$2.5x10^{-3}$	0.0	0.85
	Neuronal		1.39	0.57 to 2.21	$8.7x10^{-4}$	0.0	0.93
	Non-neuronal		0.92	0.32 to 1.51	$2.5x10^{-3}$	0.0	0.85
	Non-deprivation		0.75	0.08 to 1.42	0.03	0.0	0.96
	Body fat percentage		0.32	-0.86 to 1.50	0.59	0.0	0.94
	Non-deprivation		0.05	-1.20 to 1.30	0.94	0.0	0.95
	Waist circumference		1.32	0.58 to 2.06	$4.6x10^{-4}$	0.0	0.31
	Non-deprivation		1.35	0.54 to 2.17	$1.1x10^{-3}$	0.5	0.17

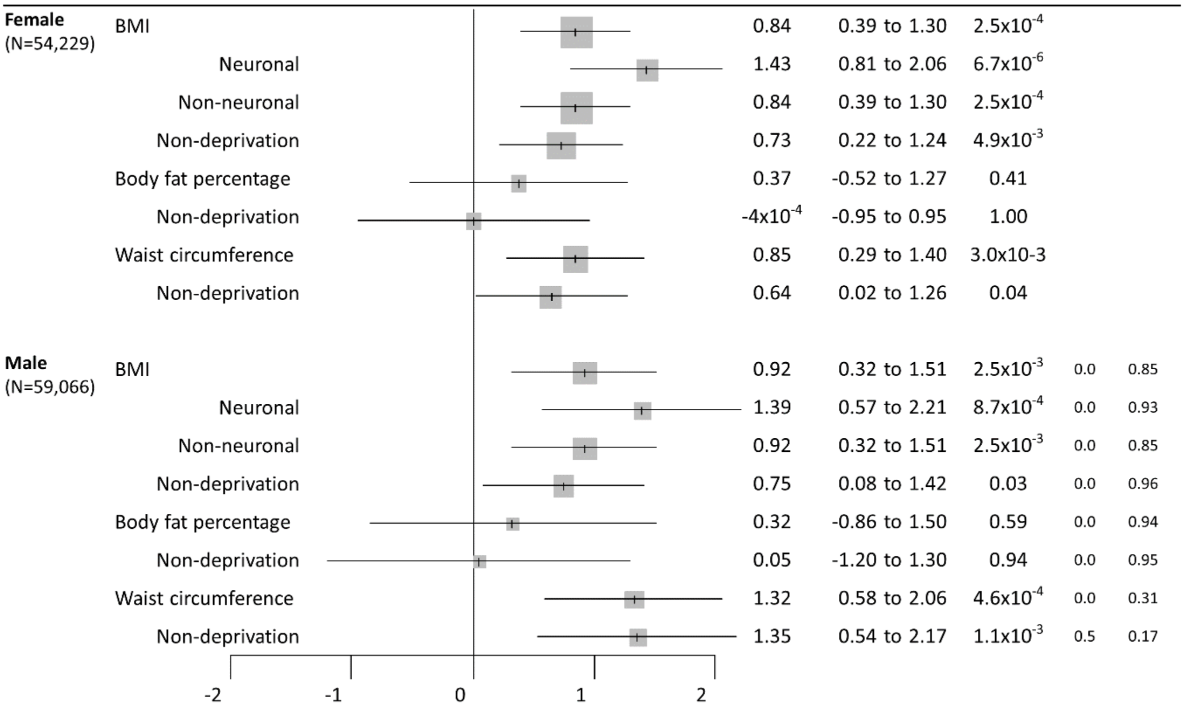

Supplementary figure U- Association between genetically determined 1 standard deviation of adiposity parameters and years of age of smoking initiation (log-transformed) in UK Biobank ever smokers stratified by sex. CI: Confidence interval. P: P value.

Sample group	Adiposity parameter	Years of age of smoking initiation (log-transformed)	95% CI	P	I^2(%)	Phet
Female (N=54,401)	BMI	-0.01	-0.02 to $1.4x10^{-3}$	0.09		
	Neuronal	-0.01	-0.03 to 0.01	0.18		
	Non-neuronal	-0.01	-0.02 to $1.4x10^{-3}$	0.09		
	Non-deprivation	$-1.5x10^{-3}$	-0.01 to 0.01	0.82		
	BF%	-0.02	-0.05 to $-2.0x10^{-3}$	0.03		
	Non-deprivation	-0.02	-0.04 to $4.0x10^{-3}$	0.10		
	WC	-0.01	-0.03 to $3.2x10^{-3}$	0.13		
	Non-deprivation	-0.01	-0.02 to 0.01	0.39		
Male (N=65,538)	BMI	-0.01	-0.02 to $1.4x10^{-3}$	0.09	0.0	0.92
	Neuronal	-0.01	-0.02 to $5.0x10^{-3}$	0.20	0.0	0.91
	Non-neuronal	-0.01	-0.02 to $1.4x10^{-3}$	0.09	0.0	0.92
	Non-deprivation	$-1.4x10^{-3}$	-0.01 to 0.01	0.82	0.0	0.99
	BF%	$1.0x10^{-3}$	-0.02 to 0.02	0.92	0.6	0.10
	Non-deprivation	$4.1x10^{-3}$	-0.02 to 0.03	0.72	0.5	0.15
	WC	-0.02	-0.03 to -0.01	$1.4x10^{-3}$	0.1	0.28
	Non-deprivation	-0.02	-0.03 to $-4.2x10^{-3}$	0.01	0.2	0.28

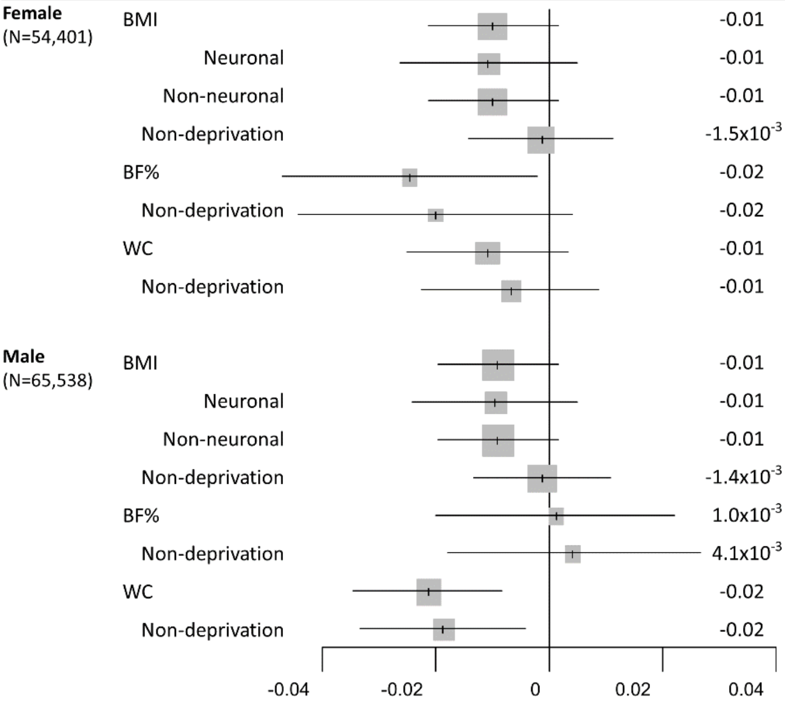

Supplementary tables

Supplementary table A – SNP parameters and summary statistics describing their association with the exposure and the outcomes.

											Association parameters to outcome															
											UK Biobank		TAG consortium													
									Association parameters to exposure		Ever vs Never smoker		Former vs Current smokers		CPD ever smokers		Age of smoking initiation*		Ever vs Never smoker		Former vs Current smokers		CPD ever smokers		Age of smoking initiation*	
SNP	Chr	Bp	Info	Adiposity parameter	Neuronal pathway	Tdi P Value	Effect allele	Other allele	Beta	SE	Beta	SE	Beta	SE	Beta	SE	Beta	SE	Beta	SE	Beta	SE	Beta	SE	Beta	SE
rs657452	1	49,589,847	0.99	BMI	-	0.04	A	G	0.023	0.003	0.015	0.005	-0.020	0.009	0.081	0.042	-0.001	0.001	0.020	0.012	0.012	0.016	0.095	0.084	0.000	0.002
rs3101336	1	72,751,185	1.00	BMI	Yes	0.48	C	T	0.033	0.003	-0.009	0.005	-0.033	0.008	-0.111	0.042	0.000	0.001	0.029	0.012	0.048	0.017	0.100	0.085	0.000	0.002
rs12566985	1	75,002,193	0.99	BMI	-	3.0E-04	G	A	0.024	0.003	0.026	0.005	-0.002	0.008	0.071	0.041	-0.003	0.001	0.017	0.012	-0.012	0.016	0.066	0.085	-0.007	0.002
rs12401738	1	78,446,761	1.00	BMI	Yes	0.05	A	G	0.021	0.003	-0.002	0.005	0.019	0.009	-0.015	0.042	0.002	0.001	-0.004	0.012	0.007	0.017	0.213	0.086	-0.007	0.002
rs11165643	1	96,924,097	1.00	BMI	Yes	0.58	T	C	0.022	0.003	-0.019	0.005	-0.010	0.008	0.018	0.042	0.001	0.001	0.013	0.012	-0.017	0.016	-0.044	0.083	0.004	0.002
rs17024393	1	110,154,688	0.99	BMI	-	0.12	C	T	0.066	0.009	-0.028	0.015	-0.022	0.026	-0.142	0.128	0.000	0.003	0.003	0.035	-0.023	0.045	0.194	0.232	-0.003	0.006
rs543874	1	177,889,480	1.00	BMI	-	2.8E-04	G	A	0.048	0.004	-0.015	0.006	0.016	0.010	-0.058	0.050	0.000	0.001	-0.001	0.015	-0.028	0.021	0.223	0.107	-0.002	0.003
rs2820292	1	201,784,287	1.00	BMI	Yes	0.02	C	A	0.020	0.003	-0.003	0.005	0.014	0.008	0.041	0.041	0.001	0.001	-0.002	0.012	-0.016	0.016	0.190	0.082	0.000	0.002
rs10182181	2	25,150,296	1.00	BMI	Yes	0.33	G	A	0.031	0.003	-0.020	0.005	-0.003	0.008	-0.034	0.041	-0.001	0.001	0.025	0.012	0.002	0.016	0.088	0.081	0.004	0.002
rs11126666	2	26,928,811	1.00	BMI	Yes	0.65	A	G	0.021	0.003	0.004	0.005	0.013	0.010	0.023	0.047	0.001	0.001	-0.021	0.013	-0.005	0.018	0.053	0.092	0.001	0.003
rs1016287	2	59,305,625	0.99	BMI	-	0.44	T	C	0.023	0.003	0.005	0.005	-0.003	0.009	-0.060	0.044	0.000	0.001	-0.013	0.013	0.009	0.017	0.055	0.090	-0.002	0.003
rs11688816	2	63,053,048	0.98	BMI	-	0.73	G	A	0.017	0.003	-0.014	0.005	0.002	0.008	0.033	0.041	0.002	0.001	-0.020	0.012	0.010	0.016	0.067	0.082	0.002	0.002
rs2121279	2	143,043,285	0.99	BMI	-	0.35	T	C	0.025	0.004	0.013	0.007	-0.005	0.013	0.030	0.062	0.000	0.001	-0.030	0.017	0.025	0.024	0.044	0.121	0.000	0.003
rs1528435	2	181,550,962	1.00	BMI	-	0.10	T	C	0.018	0.003	-0.002	0.005	-0.002	0.009	-0.039	0.042	0.000	0.001	-0.001	0.012	-0.002	0.017	0.046	0.085	0.002	0.002
rs7599312	2	213,413,231	0.97	BMI	Yes	0.55	G	A	0.022	0.003	0.000	0.005	0.012	0.009	0.027	0.046	0.000	0.001	0.018	0.013	-0.016	0.018	-0.023	0.092	0.003	0.003
rs6804842	3	25,106,437	0.99	BMI	Yes	0.83	G	A	0.019	0.003	-0.018	0.005	-0.004	0.008	-0.066	0.041	0.001	0.001	-0.007	0.013	-0.015	0.017	0.028	0.088	-0.003	0.002
rs2365389	3	61,236,462	0.99	BMI	-	0.29	C	T	0.020	0.003	0.005	0.005	-0.010	0.009	-0.024	0.042	-0.002	0.001	-0.002	0.012	0.022	0.016	0.124	0.083	0.002	0.002
rs3849570	3	81,792,112	0.99	BMI	Yes	0.11	A	C	0.019	0.003	0.007	0.005	-0.006	0.009	-0.011	0.043	0.003	0.001	-0.004	0.012	-0.039	0.017	-0.185	0.086	0.004	0.002
rs13078960	3	85,807,590	0.99	BMI	Yes	0.17	G	T	0.030	0.004	-0.037	0.006	-0.011	0.010	0.065	0.051	-0.001	0.001	0.015	0.015	0.040	0.020	-0.095	0.103	-0.001	0.003
rs16851483	3	141,275,436	1.00	BMI	-	0.66	T	G	0.048	0.008	0.000	0.009	0.014	0.017	0.014	0.082	0.002	0.002	-0.027	0.024	-0.018	0.032	-0.054	0.166	-0.002	0.005
rs1516725	3	185,824,004	0.99	BMI	Yes	0.15	C	T	0.045	0.005	-0.013	0.007	0.014	0.012	0.026	0.060	-0.004	0.001	0.017	0.017	-0.023	0.024	0.198	0.122	0.003	0.003
rs10938397	4	45,182,527	0.99	BMI	Yes	0.97	G	A	0.040	0.003	0.006	0.005	-0.015	0.008	-0.015	0.041	0.002	0.001	-0.003	0.012	-0.004	0.017	0.049	0.087	0.001	0.002
rs13107325	4	103,188,709	0.99	BMI	-	0.13	T	C	0.048	0.007	0.011	0.009	-0.052	0.016	-0.263	0.078	0.003	0.002	0.020	0.027	0.052	0.037	-0.063	0.176	0.002	0.006
rs11727676	4	145,659,064	1.00	BMI	-	0.14	T	C	0.036	0.006	0.007	0.008	0.005	0.014	0.091	0.069	0.001	0.001	-0.022	0.041	-0.001	0.051	0.027	0.288	0.003	0.006
rs2112347	5	75,015,242	1.00	BMI	-	0.55	T	G	0.026	0.003	0.007	0.005	-0.032	0.009	0.030	0.043	0.001	0.001	0.014	0.012	-0.005	0.017	0.091	0.086	0.001	0.002
rs205262	6	34,563,164	1.00	BMI	-	1.1E-03	G	A	0.022	0.004	-0.013	0.005	0.021	0.009	-0.021	0.046	0.001	0.001	0.012	0.013	0.003	0.017	0.001	0.090	-0.002	0.003
rs2207139	6	50,845,490	1.00	BMI	-	0.05	G	A	0.045	0.004	-0.005	0.006	-0.021	0.011	-0.045	0.054	0.003	0.001	0.026	0.016	0.030	0.021	0.127	0.109	-0.002	0.003
rs9400239	6	108,977,663	0.99	BMI	Yes	0.64	C	T	0.019	0.003	-0.018	0.005	0.003	0.009	0.051	0.045	0.003	0.001	0.026	0.013	0.011	0.017	-0.057	0.089	-0.002	0.002
rs13191362	6	163,033,350	0.99	BMI	Yes	0.78	A	G	0.028	0.005	-0.002	0.007	-0.015	0.013	0.008	0.062	0.002	0.001	0.022	0.018	-0.027	0.025	0.249	0.130	0.008	0.004
rs1167827	7	75,163,169	1.00	BMI	Yes	9.4E-04	G	A	0.020	0.003	-0.008	0.005	0.011	0.008	-0.092	0.041	0.000	0.001	0.014	0.013	-0.010	0.017	0.184	0.088	-0.002	0.002
rs2245368	7	76,608,143	1.00	BMI	-	0.87	C	T	0.032	0.006	0.005	0.006	-0.005	0.011	-0.088	0.055	0.000	0.001	0.005	0.035	0.030	0.041	-0.174	0.236	-0.013	0.008
rs17405819	8	76,806,584	1.00	BMI	Yes	0.62	T	C	0.022	0.003	-0.006	0.005	0.025	0.009	0.021	0.045	-0.001	0.001	0.000	0.013	0.004	0.017	-0.043	0.089	0.005	0.003
rs2033732	8	85,079,709	1.00	BMI	-	0.48	C	T	0.019	0.004	0.005	0.005	-0.002	0.010	0.013	0.047	0.000	0.001	-0.001	0.014	-0.008	0.019	-0.086	0.096	0.001	0.003
rs4740619	9	15,634,326	1.00	BMI	Yes	0.12	T	C	0.018	0.003	-0.009	0.005	-0.015	0.008	0.093	0.041	0.000	0.001	0.001	0.012	-0.026	0.016	0.034	0.082	-0.002	0.002
rs10968576	9	28,414,339	1.00	BMI	Yes	0.78	G	A	0.025	0.003	0.003	0.005	-0.002	0.009	-0.085	0.043	0.000	0.001	-0.001	0.012	0.012	0.017	-0.073	0.089	-0.001	0.003
rs6477694	9	111,932,342	0.99	BMI	-	0.22	C	T	0.017	0.003	0.007	0.005	0.003	0.009	0.056	0.043	0.000	0.001	-0.011	0.012	-0.037	0.017	0.033	0.085	-0.003	0.002
rs1928295	9	120,378,483	1.00	BMI	-	0.07	T	C	0.019	0.003	0.011	0.005	-0.011	0.008	-0.009	0.041	-0.001	0.001	-0.003	0.012	-0.002	0.016	0.110	0.081	-0.001	0.002
rs10733682	9	129,460,914	0.96	BMI	Yes	0.76	A	G	0.017	0.003	0.000	0.005	0.001	0.008	0.023	0.041	-0.001	0.001	-0.006	0.012	-0.018	0.016	0.096	0.082	-0.002	0.002

rs7899106	10	87,410,904	0.99	BMI	Yes	0.19	G	A	0.040	0.007	0.003	0.011	-0.011	0.019	0.084	0.094	0.001	0.002	0.011	0.028	-0.027	0.038	-0.001	0.195	-0.008	0.006
rs17094222	10	102,395,440	0.97	BMI	Yes	0.74	C	T	0.025	0.004	-0.009	0.006	-0.008	0.010	-0.149	0.050	0.000	0.001	-0.007	0.015	0.018	0.021	-0.137	0.106	0.000	0.003
rs11191560	10	104,869,038	1.00	BMI	-	0.04	C	T	0.031	0.005	-0.010	0.009	0.030	0.016	0.173	0.076	-0.002	0.002	0.029	0.021	-0.005	0.028	-0.016	0.144	-0.001	0.004
rs7903146	10	114,758,349	1.00	BMI	-	0.77	C	T	0.023	0.003	-0.002	0.005	0.009	0.009	0.112	0.045	0.001	0.001	0.006	0.013	0.011	0.017	-0.047	0.089	0.000	0.003
rs2316901	11	8,679,016	0.98	BMI	-	0.66	G	A	0.021	0.003	-0.010	0.005	-0.015	0.009	-0.058	0.043	0.000	0.001	-0.008	0.012	0.004	0.017	-0.158	0.084	0.002	0.002
rs11030104	11	27,684,517	1.00	BMI	Yes	0.01	A	G	0.041	0.004	0.038	0.006	-0.010	0.010	0.071	0.051	-0.002	0.001	0.052	0.014	0.001	0.019	0.063	0.100	-0.003	0.003
rs2176598	11	43,864,278	1.00	BMI	-	0.44	T	C	0.020	0.004	-0.006	0.005	-0.005	0.010	-0.016	0.047	0.000	0.001	-0.011	0.014	0.018	0.019	0.005	0.095	0.002	0.003
rs3817334	11	47,650,993	1.00	BMI	-	0.03	T	C	0.026	0.003	0.007	0.005	-0.021	0.008	0.028	0.042	0.000	0.001	-0.006	0.012	0.038	0.016	-0.053	0.083	0.003	0.002
rs12286929	11	115,022,404	0.99	BMI	Yes	0.52	G	A	0.022	0.003	0.006	0.005	0.012	0.008	-0.078	0.041	0.001	0.001	-0.017	0.012	0.012	0.016	0.060	0.082	0.001	0.002
rs7138803	12	50,247,468	1.00	BMI	Yes	0.43	A	G	0.032	0.003	0.005	0.005	-0.017	0.009	0.012	0.042	0.000	0.001	0.008	0.012	-0.022	0.016	0.021	0.084	-0.001	0.002
rs11057405	12	122,781,897	1.00	BMI	Yes	0.52	G	A	0.031	0.006	0.009	0.008	-0.006	0.014	-0.025	0.066	0.001	0.001	0.026	0.024	-0.009	0.033	-0.130	0.160	-0.005	0.005
rs9581854	13	28,017,782	0.99	BMI	-	0.72	T	C	0.030	0.005	-0.008	0.006	0.010	0.011	-0.073	0.053	0.000	0.001	0.030	0.014	0.007	0.019	-0.326	0.100	0.005	0.003
rs12429545	13	54,102,206	0.98	BMI	-	0.07	A	G	0.033	0.005	0.009	0.007	-0.002	0.013	0.021	0.061	-0.002	0.001	-0.013	0.018	0.009	0.026	-0.166	0.129	0.003	0.003
rs10132280	14	25,928,179	0.97	BMI	Yes	0.39	C	A	0.023	0.003	-0.002	0.005	-0.002	0.009	0.015	0.045	0.000	0.001	0.003	0.013	-0.016	0.018	0.131	0.093	0.000	0.003
rs12885454	14	29,736,838	1.00	BMI	Yes	0.28	C	A	0.021	0.003	0.007	0.005	0.002	0.009	0.103	0.043	0.002	0.001	0.017	0.012	-0.003	0.017	0.104	0.086	0.002	0.002
rs11847697	14	30,515,112	1.00	BMI	-	0.71	T	C	0.049	0.008	-0.002	0.011	0.040	0.020	-0.001	0.100	0.000	0.002	-0.009	0.032	0.039	0.043	0.295	0.218	-0.008	0.006
rs7141420	14	79,899,454	0.98	BMI	Yes	0.48	T	C	0.024	0.003	0.006	0.005	-0.004	0.008	0.004	0.041	0.001	0.001	-0.005	0.012	0.033	0.016	-0.041	0.081	0.001	0.002
rs3736485	15	51,748,610	0.99	BMI	Yes	0.26	A	G	0.018	0.003	-0.009	0.005	-0.010	0.008	0.017	0.041	0.000	0.001	0.002	0.012	-0.007	0.016	-0.001	0.083	-0.001	0.002
rs16951275	15	68,077,168	1.00	BMI	Yes	1.6E-05	T	C	0.031	0.004	0.021	0.006	-0.004	0.010	-0.011	0.049	-0.002	0.001	0.039	0.014	0.015	0.019	0.041	0.098	0.003	0.003
rs758747	16	3,627,358	0.98	BMI	-	0.56	T	C	0.023	0.004	0.007	0.005	-0.002	0.009	-0.035	0.046	0.001	0.001	0.000	0.014	-0.016	0.019	-0.036	0.096	0.002	0.003
rs12446632	16	19,935,389	1.00	BMI	Yes	0.14	G	A	0.040	0.005	-0.008	0.007	-0.021	0.012	0.014	0.058	0.000	0.001	-0.005	0.017	-0.028	0.023	-0.105	0.115	-0.003	0.003
rs3888190	16	28,889,486	1.00	BMI	Yes	0.02	A	C	0.031	0.003	0.003	0.005	0.020	0.008	-0.101	0.042	0.002	0.001	-0.006	0.012	-0.006	0.016	0.061	0.084	-0.001	0.002
rs9925964	16	31,129,895	1.00	BMI	Yes	4.5E-04	A	G	0.019	0.003	0.016	0.005	-0.012	0.009	0.076	0.043	-0.001	0.001	-0.006	0.012	0.001	0.016	0.194	0.084	-0.002	0.002
rs1421085	16	53,800,954	1.00	BMI	-	0.51	C	T	0.081	0.003	-0.011	0.005	-0.004	0.008	0.087	0.042	0.000	0.001	0.002	0.015	0.019	0.019	0.037	0.100	-0.002	0.002
rs1000940	17	5,283,252	0.99	BMI	-	0.35	G	A	0.019	0.003	-0.009	0.005	-0.005	0.009	0.002	0.045	0.001	0.001	0.025	0.013	-0.015	0.018	-0.046	0.090	0.000	0.003
rs12940622	17	78,615,571	1.00	BMI	-	0.49	G	A	0.018	0.003	0.007	0.005	-0.001	0.008	0.001	0.041	0.000	0.001	0.005	0.012	-0.016	0.016	0.127	0.083	-0.004	0.002
rs1808579	18	21,104,888	0.99	BMI	Yes	0.25	C	T	0.017	0.003	-0.013	0.005	-0.018	0.008	0.074	0.041	-0.001	0.001	-0.011	0.012	-0.005	0.016	0.078	0.082	-0.002	0.002
rs7243357	18	56,883,319	0.99	BMI	Yes	0.55	T	G	0.022	0.004	0.007	0.006	0.014	0.011	0.009	0.054	0.003	0.001	0.002	0.016	-0.015	0.021	0.120	0.109	-0.002	0.003
rs6567160	18	57,829,135	0.99	BMI	Yes	0.64	C	T	0.056	0.004	-0.001	0.006	0.013	0.010	-0.148	0.048	-0.003	0.001	-0.013	0.014	0.011	0.019	0.135	0.099	0.003	0.003
rs17724992	19	18,454,825	0.99	BMI	-	0.07	A	G	0.019	0.004	0.019	0.005	-0.017	0.010	0.121	0.046	0.000	0.001	0.031	0.014	0.020	0.019	-0.153	0.096	-0.001	0.003
rs29941	19	34,309,532	1.00	BMI	Yes	3.3E-03	G	A	0.018	0.003	-0.015	0.005	-0.001	0.009	-0.107	0.044	0.001	0.001	0.026	0.012	0.007	0.017	-0.067	0.088	-0.001	0.002
rs2075650	19	45,395,619	1.00	BMI	Yes	0.44	A	G	0.026	0.005	0.002	0.007	-0.025	0.012	0.025	0.058	0.000	0.001	0.011	0.021	-0.009	0.030	0.053	0.141	-0.005	0.004
rs2287019	19	46,202,172	0.98	BMI	Yes	0.99	C	T	0.036	0.004	-0.001	0.006	0.028	0.011	0.091	0.053	-0.002	0.001	-0.038	0.019	0.024	0.027	0.240	0.126	-0.004	0.004
rs3810291	19	47,569,003	1.00	BMI	Yes	0.43	A	G	0.028	0.004	-0.024	0.005	-0.002	0.009	-0.001	0.044	0.000	0.001	0.024	0.014	0.008	0.019	0.030	0.096	-0.001	0.003
rs543874	1	177,889,480	1.00	BF%	-	2.8E-04	G	A	0.031	0.006	-0.015	0.006	0.016	0.010	-0.058	0.050	0.000	0.001	-0.001	0.015	-0.028	0.021	0.223	0.107	-0.002	0.003
rs6755502	2	635,721	1.00	BF%	-	0.15	C	T	0.035	0.006	-0.035	0.006	0.005	0.011	-0.140	0.055	0.003	0.001	0.036	0.015	-0.039	0.021	0.112	0.107	-0.003	0.003
rs6738627	2	165,544,450	1.00	BF%	-	0.18	A	G	0.030	0.005	0.000	0.005	-0.007	0.009	0.073	0.043	0.001	0.001	-0.025	0.020	-0.039	0.023	-0.229	0.126	0.003	0.003
rs2943652	2	227,108,446	1.00	BF%	-	0.24	C	T	0.030	0.005	0.003	0.005	-0.007	0.009	0.008	0.043	0.001	0.001	0.011	0.012	0.011	0.016	0.068	0.084	0.002	0.002
rs693839	13	80,958,288	0.99	BF%	-	0.15	C	T	0.030	0.005	0.008	0.005	-0.015	0.009	-0.007	0.045	0.000	0.001	0.016	0.012	0.035	0.017	-0.012	0.087	0.003	0.002
rs4788099	16	28,855,727	1.00	BF%	-	0.02	G	A	0.026	0.005	0.003	0.005	0.020	0.008	-0.099	0.042	0.002	0.001	-0.006	0.012	-0.007	0.016	0.065	0.084	-0.001	0.002
rs1421085	16	53,800,954	1.00	BF%	-	0.51	C	T	0.051	0.005	-0.011	0.005	-0.004	0.008	0.087	0.042	0.000	0.001	0.002	0.015	0.019	0.019	0.037	0.100	-0.002	0.002
rs9906944	17	47,091,420	0.99	BF%	-	0.32	C	T	0.035	0.006	0.009	0.005	-0.022	0.009	-0.009	0.043	-0.002	0.001	-0.002	0.013	-0.016	0.018	0.104	0.091	0.003	0.003
rs6567160	18	57,829,135	0.99	BF%	-	0.64	C	T	0.030	0.006	-0.001	0.006	0.013	0.010	-0.148	0.048	-0.003	0.001	-0.013	0.014	0.011	0.019	0.135	0.099	0.003	0.003
rs757318	19	18,820,308	1.00	BF%	-	0.09	C	A	0.024	0.005	0.007	0.005	-0.005	0.008	0.054	0.041	-0.001	0.001	0.012	0.012	0.008	0.017	0.111	0.085	-0.001	0.002
rs6857	19	45,392,254	1.00	BF%	-	0.19	C	T	0.053	0.009	0.003	0.006	-0.024	0.011	0.012	0.054	0.000	0.001	0.015	0.021	-0.018	0.030	0.041	0.141	-0.003	0.004
rs3761445	22	38,595,411	1.00	BF%	-	0.19	G	A	0.024	0.005	0.003	0.005	0.000	0.009	-0.031	0.042	0.001	0.001	-0.021	0.012	0.009	0.016	-0.161	0.083	0.002	0.002
rs3127553	1	49438005	1.01	WC	-	0.01	G	A	0.023	0.004	0.020	0.005	-0.028	0.009	0.064	0.042	-0.001	0.001	-0.028	0.015	0.004	0.019	-0.041	0.102	-0.001	0.002
rs7531118	1	72837239	0.97	WC	-	0.71	C	T	0.027	0.004	-0.006	0.005	-0.031	0.008	-0.114	0.041	0.000	0.001	-0.019	0.012	-0.052	0.017	-0.145	0.085	0.001	0.002
rs4130548	1	78463868	1.00	WC	-	0.06	C	T	0.022	0.004	-0.002	0.005	0.019	0.009	-0.015	0.042	0.002	0.001	0.002	0.012	0.002	0.017	-0.212	0.085	0.007	0.002
rs11165623	1	96893000	1.00	WC	-	0.86	A	G	0.020	0.003	-0.021	0.005	-0.003	0.008	0.044	0.041	-0.001	0.001	-0.003	0.012	-0.029	0.016	-0.016	0.082	0.004	0.002
rs7550711	1	110082886	0.99	WC	-	0.14	T	C	0.058	0.010	-0.033	0.015	-0.006	0.026	-0.173	0.128	-0.001	0.003	0.019	0.036	-0.041	0.047	0.139	0.243	-0.002	0.007
rs633715	1	177852580	1.00	WC	-	1.5E-03	C	T	0.043	0.004	-0.013	0.006	0.016	0.010	-0.066	0.050	-0.001	0.001	-0.009	0.016	0.034	0.021	-0.216	0.109	0.002	0.003
rs2820292	1	201784287	1.00	WC	-	0.02	C	A	0.019	0.003	-0.003	0.005	0.014	0.008	0.041	0.041	0.001	0.001	0.002	0.012	0.016	0.016	-0.190	0.082	0.000	0.002

rs6755502	2	635721	1.00	WC	-	0.15	C	T	0.051	0.005	-0.035	0.006	0.005	0.011	-0.140	0.055	0.003	0.001	-0.036	0.015	0.039	0.021	-0.112	0.107	0.003	0.003
rs929641	2	58792377	1.00	WC	-	0.03	A	G	0.021	0.003	-0.001	0.005	-0.005	0.008	0.029	0.041	-0.001	0.001	0.004	0.012	0.022	0.016	0.058	0.082	0.000	0.002
rs6545714	2	59307725	0.99	WC	-	0.67	G	A	0.022	0.004	0.019	0.005	0.000	0.008	-0.021	0.042	0.000	0.001	-0.007	0.012	-0.006	0.016	-0.113	0.083	0.002	0.002
rs3849570	3	81792112	0.99	WC	-	0.11	A	C	0.021	0.004	0.007	0.005	-0.006	0.009	-0.011	0.043	0.003	0.001	-0.004	0.012	-0.039	0.017	-0.185	0.086	0.004	0.002
rs2325036	3	85819412	0.99	WC	-	0.35	A	C	0.023	0.004	0.037	0.005	-0.015	0.009	-0.109	0.042	-0.004	0.001	0.038	0.012	0.001	0.016	-0.023	0.084	0.000	0.002
rs6440003	3	141094209	1.00	WC	-	0.72	A	G	0.022	0.003	0.001	0.005	-0.017	0.008	-0.066	0.041	-0.001	0.001	0.006	0.012	0.009	0.016	-0.028	0.083	-0.007	0.002
rs1516725	3	185824004	0.99	WC	-	0.15	C	T	0.031	0.005	-0.013	0.007	0.014	0.012	0.026	0.060	-0.004	0.001	-0.017	0.017	0.023	0.024	-0.198	0.122	-0.003	0.003
rs10938397	4	45182527	0.99	WC	-	0.97	G	A	0.032	0.004	0.006	0.005	-0.015	0.008	-0.015	0.041	0.002	0.001	0.003	0.012	0.004	0.017	-0.049	0.087	-0.001	0.002
rs2112347	5	75015242	1.00	WC	-	0.55	T	G	0.025	0.004	0.007	0.005	-0.032	0.009	0.030	0.043	0.001	0.001	0.014	0.012	-0.005	0.017	0.091	0.086	0.001	0.002
rs806794	6	26200677	1.00	WC	-	0.85	A	G	0.022	0.004	0.011	0.005	-0.014	0.009	0.174	0.046	-0.004	0.001	0.049	0.013	-0.042	0.018	0.027	0.091	0.000	0.003
rs16894959	6	34825662	1.01	WC	-	4.0E-03	C	T	0.026	0.005	-0.011	0.007	0.040	0.012	0.096	0.059	-0.001	0.001	-0.008	0.017	0.007	0.022	0.090	0.117	0.005	0.003
rs2033529	6	40348653	0.99	WC	-	0.06	G	A	0.021	0.004	-0.004	0.005	0.007	0.009	-0.056	0.045	-0.001	0.001	-0.022	0.013	-0.005	0.018	0.046	0.090	-0.005	0.003
rs943005	6	50865820	1.00	WC	-	0.05	T	C	0.039	0.004	-0.004	0.006	-0.021	0.011	-0.044	0.054	0.003	0.001	0.024	0.015	0.029	0.021	0.118	0.108	-0.002	0.003
rs9400239	6	108977663	0.99	WC	-	0.64	C	T	0.024	0.004	-0.018	0.005	0.003	0.009	0.051	0.045	0.003	0.001	-0.026	0.013	-0.011	0.017	0.057	0.089	0.002	0.002
rs2489623	6	127455821	0.99	WC	-	0.25	C	A	0.019	0.003	0.006	0.005	-0.011	0.008	-0.047	0.041	0.000	0.001	0.010	0.012	-0.006	0.016	-0.126	0.083	-0.002	0.002
rs10968576	9	28414339	1.00	WC	-	0.78	G	A	0.025	0.004	0.003	0.005	-0.002	0.009	-0.085	0.043	0.000	0.001	0.001	0.012	-0.012	0.017	0.073	0.089	0.001	0.003
rs6163	10	104596924	1.00	WC	-	0.01	A	C	0.019	0.004	-0.030	0.005	0.026	0.009	-0.075	0.042	0.002	0.001	0.041	0.012	0.005	0.016	0.115	0.082	-0.001	0.002
rs7903146	10	114758349	1.00	WC	-	0.77	C	T	0.022	0.004	-0.002	0.005	0.009	0.009	0.112	0.045	0.001	0.001	-0.006	0.013	-0.011	0.017	0.047	0.089	0.000	0.003
rs10840100	11	8669437	1.00	WC	-	0.67	G	A	0.020	0.004	0.010	0.005	0.015	0.009	0.060	0.043	0.000	0.001	0.008	0.012	-0.004	0.016	0.156	0.084	-0.002	0.002
rs2293576	11	47434986	1.00	WC	-	0.36	G	A	0.022	0.004	-0.002	0.005	0.022	0.009	0.110	0.043	-0.001	0.001	0.009	0.012	-0.026	0.017	0.186	0.086	0.000	0.002
rs7138803	12	50247468	1.00	WC	-	0.43	A	G	0.028	0.004	0.005	0.005	-0.017	0.009	0.012	0.042	0.000	0.001	0.008	0.012	-0.022	0.016	0.021	0.084	-0.001	0.002
rs12429545	13	54102206	0.98	WC	-	0.07	A	G	0.031	0.005	0.009	0.007	-0.002	0.013	0.021	0.061	-0.002	0.001	-0.013	0.018	0.009	0.026	-0.166	0.129	0.003	0.003
rs10132280	14	25928179	0.97	WC	-	0.39	C	A	0.022	0.004	-0.002	0.005	-0.002	0.009	0.015	0.045	0.000	0.001	-0.003	0.013	0.016	0.018	-0.131	0.093	0.000	0.003
rs12885454	14	29736838	1.00	WC	-	0.28	C	A	0.020	0.004	0.007	0.005	0.002	0.009	0.103	0.043	0.002	0.001	-0.017	0.012	0.003	0.017	-0.104	0.086	-0.002	0.002
rs7144011	14	79940383	0.99	WC	-	0.04	T	G	0.033	0.004	0.008	0.006	-0.029	0.010	0.008	0.049	0.001	0.001	0.010	0.014	0.030	0.019	-0.120	0.099	0.006	0.003
rs2531992	16	4021734	1.00	WC	-	0.31	G	A	0.028	0.005	-0.014	0.006	0.030	0.012	-0.051	0.057	0.002	0.001	-0.045	0.017	-0.046	0.024	-0.306	0.122	0.007	0.003
rs12446632	16	19935389	1.00	WC	-	0.14	G	A	0.036	0.005	-0.008	0.007	-0.021	0.012	0.014	0.058	0.000	0.001	0.005	0.017	0.028	0.023	0.105	0.115	0.003	0.003
rs7498665	16	28883241	1.00	WC	-	0.02	G	A	0.034	0.004	0.003	0.005	0.020	0.009	-0.094	0.042	0.003	0.001	0.006	0.012	0.007	0.016	-0.071	0.084	0.001	0.002
rs1549293	16	31141993	1.32	WC	-	3.9E-04	C	T	0.020	0.004	0.017	0.005	-0.012	0.009	0.081	0.043	-0.001	0.001	0.009	0.012	-0.001	0.016	-0.202	0.084	0.002	0.002
rs62048402	16	53803223	1.00	WC	-	0.50	A	G	0.074	0.004	-0.011	0.005	-0.004	0.008	0.089	0.042	0.000	0.001	0.003	0.015	0.021	0.019	-0.032	0.101	-0.001	0.002
rs7239883	18	40147671	0.99	WC	-	0.18	G	A	0.021	0.004	0.005	0.005	-0.002	0.009	0.076	0.042	-0.001	0.001	-0.013	0.012	0.006	0.016	-0.074	0.083	0.003	0.002
rs6567160	18	57829135	0.99	WC	-	0.64	C	T	0.048	0.004	-0.001	0.006	0.013	0.010	-0.148	0.048	-0.003	0.001	0.013	0.014	-0.011	0.019	-0.135	0.099	-0.003	0.003
rs2075650	19	45395619	1.18	WC	-	0.44	A	G	0.031	0.005	0.002	0.007	-0.025	0.012	0.025	0.058	0.000	0.001	0.011	0.021	-0.009	0.030	0.053	0.141	-0.005	0.004
rs2287019	19	46202172	0.98	WC	-	0.99	C	T	0.035	0.005	-0.001	0.006	0.028	0.011	0.091	0.053	-0.002	0.001	0.038	0.019	-0.024	0.027	-0.240	0.126	0.004	0.004
rs3810291	19	47569003	1.00	WC	-	0.43	A	G	0.026	0.004	-0.024	0.005	-0.002	0.009	-0.001	0.044	0.000	0.001	0.024	0.014	0.008	0.019	0.030	0.096	-0.001	0.003
rs16996700	20	50981945	1.00	WC	-	0.87	T	C	0.023	0.004	0.007	0.005	0.000	0.009	0.118	0.045	0.000	0.001	0.000	0.013	-0.054	0.018	0.054	0.090	0.000	0.003

Footnote: Chr: Chromosome. Bp: Base pair position. Info: R^2 imputation quality parameter. Tdi: Townsend deprivation index. SE: Standard error. CPD: Cigarettes smoked per day. *: log-transformed years. BMI: Body mass index. BF%: Body fat percentage. WC: Waist circumference.
